# Molecular Boronic Acid-Based Saccharide Sensors

**DOI:** 10.1021/acssensors.1c00462

**Published:** 2021-04-12

**Authors:** George T. Williams, Jonathan L. Kedge, John S. Fossey

**Affiliations:** School of Chemistry, University of Birmingham, Edgbaston, Birmingham, West Midlands, B15 2TT, United Kingdom

**Keywords:** carbohydrate, fluorescence, electrochemical, colorimetric, diabetes, biomarker, boronic acids, hydrogels, glucose

## Abstract

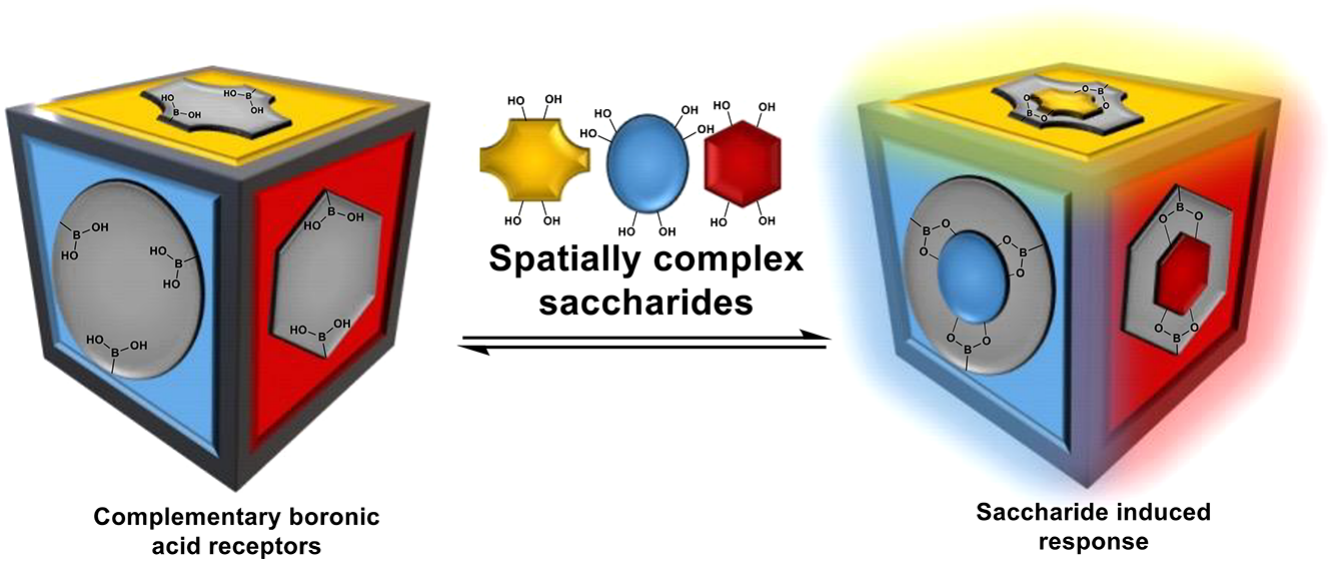

Boronic acids can reversibly bind diols, a molecular feature that is ubiquitous within saccharides, leading to their use in the design and implementation of sensors for numerous saccharide species. There is a growing understanding of the importance of saccharides in many biological processes and systems; while saccharide or carbohydrate sensing in medicine is most often associated with detection of glucose in diabetes patients, saccharides have proven to be relevant in a range of disease states. Herein the relevance of carbohydrate sensing for biomedical applications is explored, and this review seeks to outline how the complexity of saccharides presents a challenge for the development of selective sensors and describes efforts that have been made to understand the underpinning fluorescence and binding mechanisms of these systems, before outlining examples of how researchers have used this knowledge to develop ever more selective receptors.

Essential to life as we understand it, saccharides (or carbohydrates) are ubiquitous throughout the natural world. From simple monosaccharides consisting of a single unit, to complex oligosaccharides composed of hundreds or thousands of individual monomers, these molecules form a diverse range of structures allowing them to perform a wide range of diverse roles. Glucose (**1**) for example is the primary metabolic fuel, produced during photosynthesis by plants harvesting the power of sunlight.^1^ Cellulose, formed from a branched chain of multiple linked glucose molecules, provides structural rigidity to plant cell walls,^2^ while more complex oligosaccharides, consisting of numerous different saccharide monomers, are used primarily for cell-recognition events (blood-type antigens, for example, are glycolipids).^3^ Saccharides also play a key role in the storage of genetic information; a five-membered deoxyribose sugar is a key component of the polynucleotide backbone that forms DNA.^4^ The aminoglycosides are amino-modified sugars that display potent antimicrobial activity and are the antibiotic of choice for the treatment of a variety of bacterial infections.^5^ Beyond their biological applications, carbohydrates have also been investigated as renewable feedstock polymeric materials, as alternative fuels to petrochemicals.^6^

There is a great deal of chemical and spatial complexity inherent within saccharides. The structures of even simple saccharides such as glucose are governed by the presence of equilibria between cyclic and linear structures. d-Glucose (**1**) exists as a mixture (at 27 °C in D_2_O) of six-membered α-d-glucopyranose (38.8%) and β-d-glucopyranose (60.9%) rings; a small portion of the equilibrium consists of five-membered furanose rings, α-d-glucofuranose (0.14%) and β-d-glucofuranose (0.15%), Figure 1. While glucose may also exist in its acyclic form, this tends to be present in negligible quantities in aqueous solutions (0.0024%).^7^

**Figure 1:**
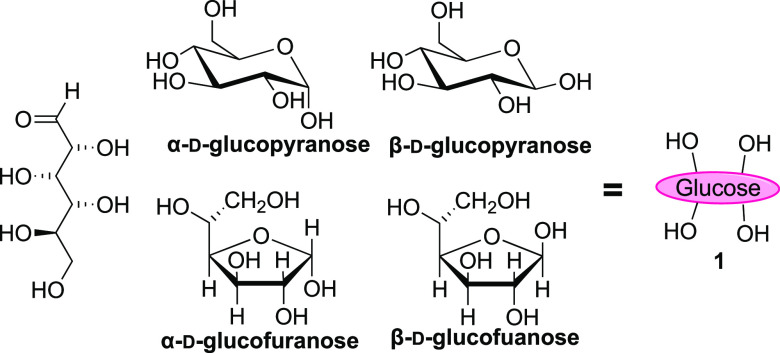
d-Glucose (**1**) in its acyclic, α-d-glucopyranose, β-d-glucopyranose, α-d-glucofuranose, and β-d-glucofuranose forms.

This inherent complexity is compounded as larger carbohydrates are considered (Figure 2), with oligosaccharides presenting a bewildering number of different isomers.^8^ Disaccharides (such as **4**, **5**, and **6**) trisaccharides (such as **7**), and tetrasaccharides (such as **9**) are two, three, and four sugar units respectively, bound through glyosidic bonds. Since this bond may theoretically occur between the anomeric carbon of one sugar and any unmodified hydroxyl group of another, a tetrasaccharide consisting of four identical glucopyranose units would exhibit a staggering 1792 individual isomers (**7**).^9^ Nature has evolved biochemical tools with which to produce exquisitely complicated structures with a high precision that our diagnostic tools are, as yet, unable to completely adequately address.

**Figure 2:**
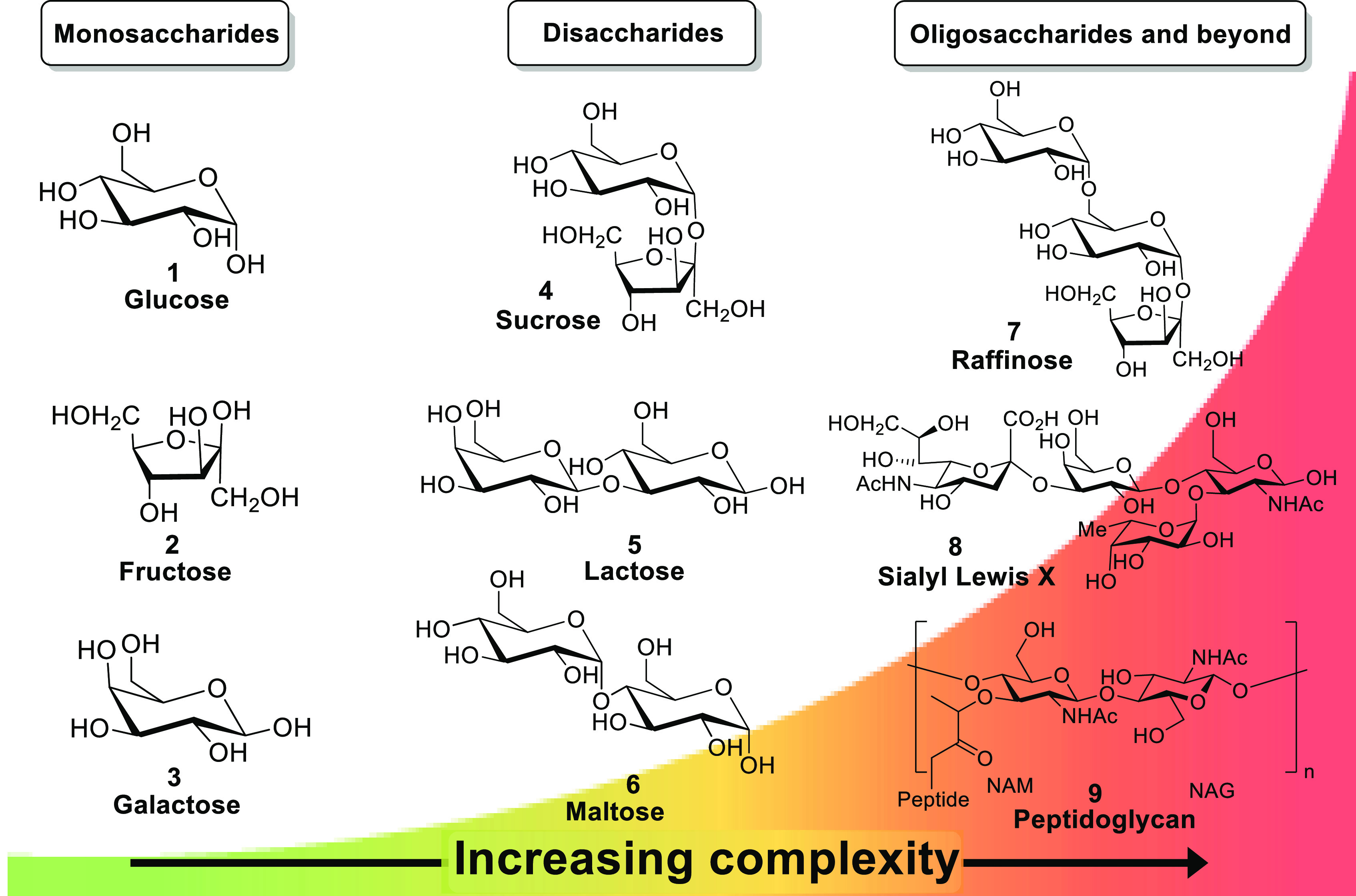
Increasing complexity of saccharides as higher order structures are formed from simple saccharides, outlining the difficulties that can be faced when selectively sensing larger saccharides.

The selective recognition and sensing of individual saccharides is challenging, especially in the context of various biological settings and physiological environments, which can present a barrier to attaining sensitivity.^10^ Solvent competition about recognition domains,^11^ especially in water, can introduce entropic and enthalpic barriers toward efficient receptor binding,^12−15^ while the presence of other saccharides may interfere with results.^16^ Blood-glucose concentrations typically lie in the range of 3.5–5.5 mM, making it the most abundant carbohydrate in blood.^17^ This is far in excess of all of the other simple saccharides with fructose (**2**), the second most concentrated saccharide, having a normal blood concentration of <0.1 mM.^18^ The low concentrations of analytes such as fructose when compared to glucose is fortuitous, given aspects of off-target selectivity that will be explored later in this review.

## Biological Importance of Saccharides

Saccharides, oligosaccharides, and their protein conjugates also play important roles in the development and progression of cancer. Metastatic tumors for example, often present abnormal *N*-glycosylation and post-transcriptional *O*-glycosylation, in which monosaccharides are covalently bound to specific proteins at asparagine, or serine and threonine residues, respectively.^19^ Not only are these glycol-related events of great importance to our understanding of cancer mechanics they also provide a means by which the disease can be detected and then monitored.^20^ Sialyl Lewis X (sLe^x^, **8**) is a cell-surface tetrasaccharide, which is overexpressed in cancerous tissues providing a good indication of malignancy.^21^ With much progress in the field of glycomics,^22,23^ glycan biomarkers are proving increasingly useful for early diagnosis as well as stratifying, staging, and monitoring of disease. Unfortunately, the antigen-based technologies currently available to detect, identify, and quantify these complex biological molecules are expensive, time-consuming to produce, and fraught with difficulties.^24^ Appropriate chemosensors for these cancer specific biomarkers, which could be relatively quickly and cheaply produced, are therefore highly sought-after.

Outside the animal kingdom, carbohydrates are essential to physiological and pathological processes in bacteria,^25−29^ viruses,^30,31^ and fungi.^32−34^ The bacterial cell wall consists of peptidoglycans (**9**), specifically *N*-acetylglucosamine (NAG) and *N*-acetylmuramic acid (NAM).^35^ These saccharide-derived polymers are essential to the survival of the bacteria, providing structural integrity and preventing osmotic lysis. It is the cell wall that differentiates Gram-positive and Gram-negative bacteria; the peptidoglycan layer is thick (≈80 nm) and porous in Gram-positive bacteria, enabling it to retain the crystal violet dye during the Gram staining test.^36^ Conversely the cell wall of Gram-negative bacteria is much thinner (1.5–10 nm),^37^ and as such the stain is not retained. To improve their survival fitness, bacteria often produce antimicrobials, many of which have been added to the anti-infective armamentarium. One such class is the previously mentioned aminoglycosides, amino sugars with broad spectrum activity against Gram-negative bacteria, as well as *Mycobacterium tuberculosis*.^38^ Viruses are also dependent on saccharide derivatives, utilizing glycoproteins (i.e., hemagglutinin) to bind to host cells.^39^ Interactions with saccharide derivatives play an important role in viral host recognition, and this has been exploited in the development of diagnostic and therapeutic technologies.^40−42^

As a set of molecules fundamental to life, the ability to selectively sense the multitude of diverse carbohydrate structures is of huge importance to modern medicine. *Diabetes mellitus*, which is characterized by the body’s inability to effectively regulate blood-glucose levels, can lead to numerous health complications including increased cardiac risk and an increased propensity for chronic wound formation.^43,44^ It presents a major health and economic crisis, affecting hundreds of millions of people worldwide at a cost of hundreds of billions of dollars.^45^ In the United States between 1990 and 2010 the number of people living with diabetes increased 3-fold, and the number of new incidences doubled. Current forecasts predict that from 2015 to 2030 the incidents of diabetes will increase by 54% while annual deaths resulting from diabetes will increase by 38%.^45^ There is no direct cure for diabetes, and as such management of this disease requires constant vigilance to prevent patients becoming hypo-/hyperglycaemic. Blood-glucose levels are invasively monitored multiple times a day by some patients; thus, a clinical need for less invasive technologies capable of continuous blood-glucose monitoring (CGM) exists.^46,47^ Molecular chemical sensors can be broadly classified into two types. *Chemosensors* reversibly bind analytes and continuously report their presence (and changes in relative amount/concentration), while *chemodosimeters* involve an irreversible chemical reaction that produces a response, giving a cumulative count of the total exposure of the reporter unit to the analyte.^48^ General dosimeters are an important class of reporter; the ability to report the cumulative dose of an analyte has been utilized within radiation badges, which are able to inform the wearer if of a cumulative exposure level rather than an instantaneous readout.^49^

Chemosensors and chemodosimeters find application in medicine and environmental analysis; their development, construction, and application have reviewed previously.^48,50−54^ Generally both molecular chemosensors and molecular chemodosimeters (including those based on boronic acid chemistry) utilize a receptor motif that detects the presence of an analyte. The recognition event then produces a chemical signal translated reversibly (sensor) or irreversibly (dosimeter) by a reporter component to a measurable output or readout, which is commonly a change in the fluorescence properties. There are multiple mechanisms by which fluorescence has been used to signal the presence of an analyte. The most common fluorescence mechanisms utilized in saccharide sensing are photoinduced electron transfer (PET), Förster resonance energy transfer (FRET), and internal charge transfer (ICT).^55,56^ A typical PET fluorescence sensing/signaling mechanism involves excitation of an analyte-free molecule which undergoes an intramolecular electron transfer from the excited fluorophore to the analyte-free receptor, preventing a fluorescence output, a fluorescence-off state. Upon the binding of an analyte to the receptor part of the molecule, the HOMO energy level of the receptor is reduced, preventing the aforementioned energy transfer, thus producing a fluorescence-on state in the presence of analyte.^57,58^ FRET involves the use of two fluorophores which act as a “donor” and “acceptor” pair that may be intra- or intermolecular in nature; when the donor–acceptor pair are within close proximity (typically around 10 nm), the fluorescence of the donor is quenched.^59^ Binding events that can change conformation or relative orientation and distance of the paired motifs can therefore be detected through resulting fluorescence modulation.^60,61^ Molecular reporting systems that utilize an ICT fluorescence mechanism are often referred to as “push–pull” systems, wherein an electron donating group and electron withdrawing group are typically positioned at opposing points on a single molecule.^55^ Modulation of the distribution of electron density through analyte binding (or irreversible reaction in the case of dosimeters),^62,63^ results in a shift of the spectral properties of the molecule which can be manifested as changes in fluorescence.

To circumvent the previously described issues pertaining to the detection and sensing of saccharides in biological environments, many glucose monitoring devices rely on a quantitative enzymatic process as a proxy for blood-glucose levels. Glucose oxidase specifically oxidizes d-glucose, yielding d-glucono-δ-lactone and commensurate amounts of hydrogen peroxide, without producing a response to other common saccharides. The hydrogen peroxide then subsequently oxidizes a reporter, producing a response, commonly either colorimetric,^64^ fluorescent,^65^ or electrochemical^66^ in nature. These chemodosimeters, while effective, are unable to act as CGMs due to the consumption of the hydrogen peroxide sensitive reporter. However, owing to their cost efficiency and ease of use, these enzyme reliant systems have been a mainstay of the commercial market for glucose sensing.^67^ Alternatively, there are synthetic chemosensors that detect saccharides directly, which may find applications in CGMs. These for the most part can be classified into two types. The first is to exploit noncovalent interactions such as hydrogen bonding and π-stacking to encapsulate their polyhydroxylated saccharide targets, of which molecular “temples” pioneered by Davis and co-workers provide an exquisite example.^68−71^ The second, upon which this review will focus, relies upon the interaction between boronic acid derivatives and diols contained within saccharides, the so-called *boronolectins*.^72,73^

## Boronic Acid-Based Saccharide Sensor Design

**Scheme 1:**
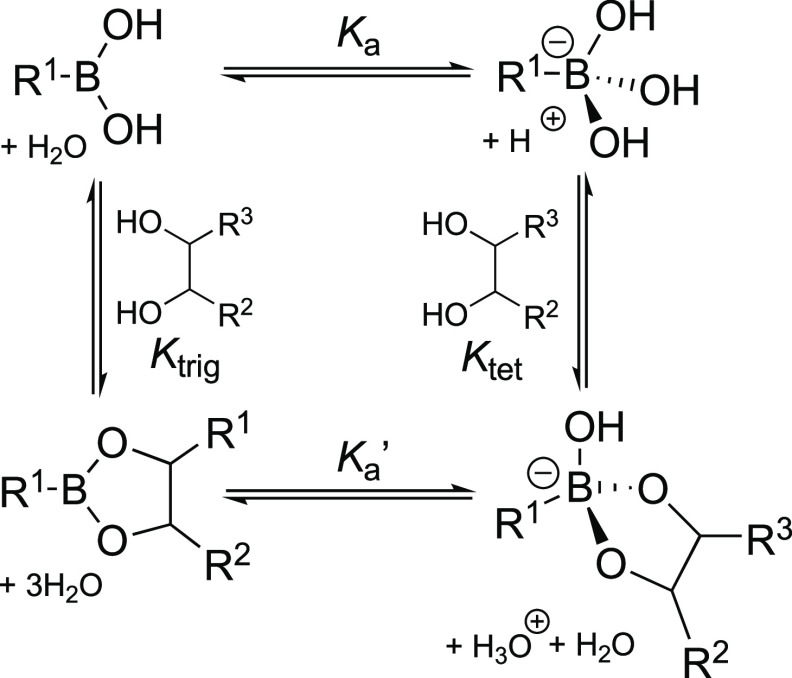
Equilibria between Boronic Acid and Ester Derivatives in the Presence of 1,2-Diols

As first reported in detail by Lorand and Edwards,^74^ oxygens of alcohols are capable of forming dynamic covalent B–O bonds with the boron of boronic acid derivatives, resulting in the formation of boronic or boronate esters. Due to both the geometry and valency of boron, the *cis*-1,2- and -1,3-diols, ubiquitous to saccharides, form particularly stable five- and six-membered cyclic boronic esters, respectively, Scheme 1.^74^

Unfortunately, for most boronic acid derivatives, the formation of boronic esters is slow at physiological pH.^75^ The predominating neutral, trigonal boronic acid does not readily exchange ligands and is therefore impractical for sensing applications. It has however been observed that the incorporation of an appropriately placed basic amino group lowers the p*K*_a_ of the acid, shifting the equilibrium in favor of the charged boronate form, thus facilitating rapid ligand exchange.^76^

**Scheme 2:**
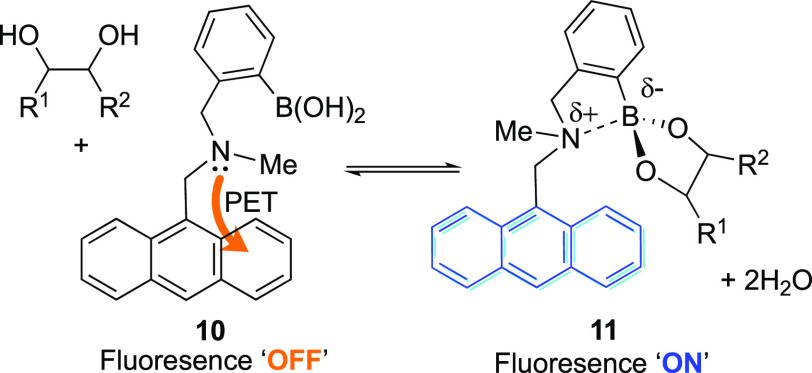
PET Mechanism of Fluorescence Modulation Proposed by Shinkai and Co-workers^77^

In 1994, Shinkai and co-workers augmented a boronic acid-binding motif with an anthracene reporter group to produce the first fluorescent (*o*-(aminomethyl)phenyl)boronic acid saccharide sensor, **10**, which reversibly binds to diols to form boronic ester **11**.^77^ At the time, in accordance with other reports, a PET mechanism seemed the most likely explanation for the observed changes in fluorescence, Scheme 2.^78^ It was proposed that, in the absence of analyte, the N–B interaction was weak and the lone pair on nitrogen available to quench the fluorophore’s fluorescence. Upon binding a saccharide, however, the acidity of the boron increases, producing a stronger Lewis interaction with the amine’s lone pair, reducing its quenching effects and revealing the inherent fluorescence of anthracene.

Like other monoboronic acid derivatives, the system proposed by Shinkai and co-workers is strongly selective for d-fructose (**2**, *K*_a_ = 1000 M^–1^) over d-glucose (*K*_d_ = 63 M^–1^) and the other biologically relevant saccharide d-galactose (*K*_a_ = 158 M^–1^) (as measured in 33 wt % aqueous MeOH buffer at pH 7.77).^74,79^ This selectivity is usually attributed to the *syn*-periplanar-1,2-diol motif present in the β-d-furanose form of glucose which comprises ∼25% of the equilibrium mixture (D_2_O, 31 °C).^14,80^ Although the α-d-furanose form of glucose also bears a *syn*-periplanar-1,2-diol, this is a minor component in an aqueous environment comprising just ∼0.14% of the equilibrium mixture (D_2_O, 27 °C).^7^

**Scheme 3:**
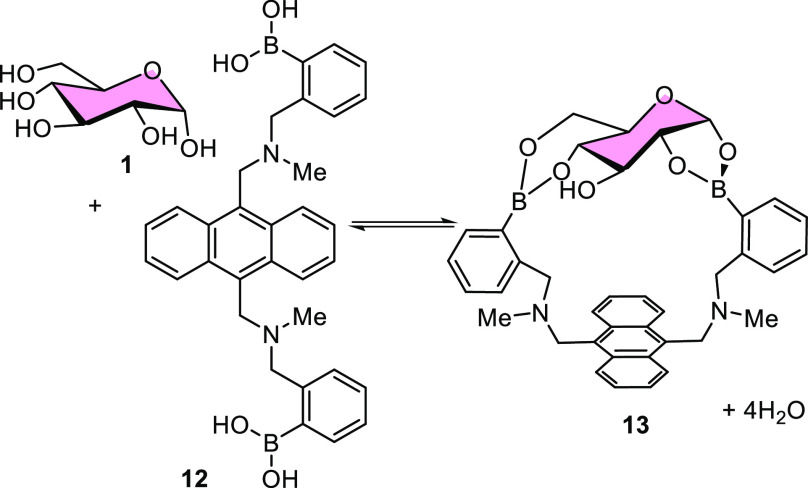
Bidentate Binding of the Glucose-Selective Bis-boronic Acid Sensor **12** Designed by James et al.^82^

Importantly, glucose bears multiple diol motifs and thus affords the possibility of stronger, ditopic binding to two boronic acid motifs. This opportunity was realized by James and co-workers who produced the first glucose-selective bisboronic acid **12**, Scheme 3. Incredibly, the observed glucose stability constant for this system (*K*_a_ = 3981 M^–1^) was over an order of magnitude greater than that of fructose (**2**, *K*_a_ = 316 M^–1^) and galactose (*K*_d_ = 158 M^–1^) (33 wt % MeOH in aqueous buffer at pH 7.77).^79^ As might be expected from a multivalent binding motif, the spacing between the boronic acid units greatly influences the strength of glucose binding. Exploring a modular series of bis-boronic acids, James et al. demonstrated that optimization of linker length between the two boronic acid motifs was crucial for achieving high glucose selectivity, **13**. For example, a six-carbon linker separating amino boronic acid motifs conferred a 3-fold increase in glucose binding constant (*K*_d_ = 962 M^–1^) compared to five (*K*_d_ = 333 M^–1^) or seven (*K*_d_ = 336 M^–1^) carbon linked congeners.^81^

**Scheme 4:**
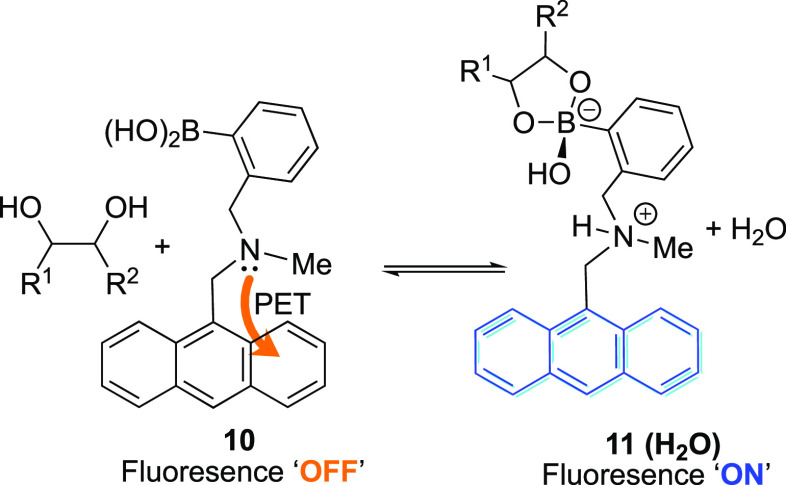
Solvent Insertion Mechanism for Emission Turn-On of (*o*-Aminomethylphenyl)boronic Acid-Based Saccharide Sensors As Investigated Separately by Wang and Co-workers,^83,84^ Anslyn and Co-workers,^76^ and Larkin et al.^85,86^

Since the landmark discovery of glucose-selective binding resulting from bespoke bis-boronic acid derivatives, interest in these systems has grown. However, as understanding has developed, discrepancies in aspects of the PET signaling model have been revealed; in response, more nuanced analyses of the mechanism have evolved. Wang and co-workers, for instance, used DFT calculations to predict that an N–B bond is in fact weaker in the boronic ester than the corresponding boronic acid and, hence, could not fully account for the observed fluorescence turn-on. Instead they proposed that in protic solvents a “p*K*_a_-switch” dominates, in which the formation of the boronate ester results in solvent insertion (Scheme 4), leading to hydrolysis of the weak N–B bond with subsequent protonation of the amine, which thus shuts down the PET quenching pathway.^83,84^ This conclusion was further supported by Collins et al. through the use of ^11^B NMR and X-ray crystal structure studies.^76^ The nature of the N–B bond was also studied by Larkin et al., who probed a range of *o*-((*N*,*N*-dialkylamino)methyl)arylboronates computationally and determined that the an N–HO–B interaction can predominate over a direct N–B bond in some cases.^85,86^

**Scheme 5:**
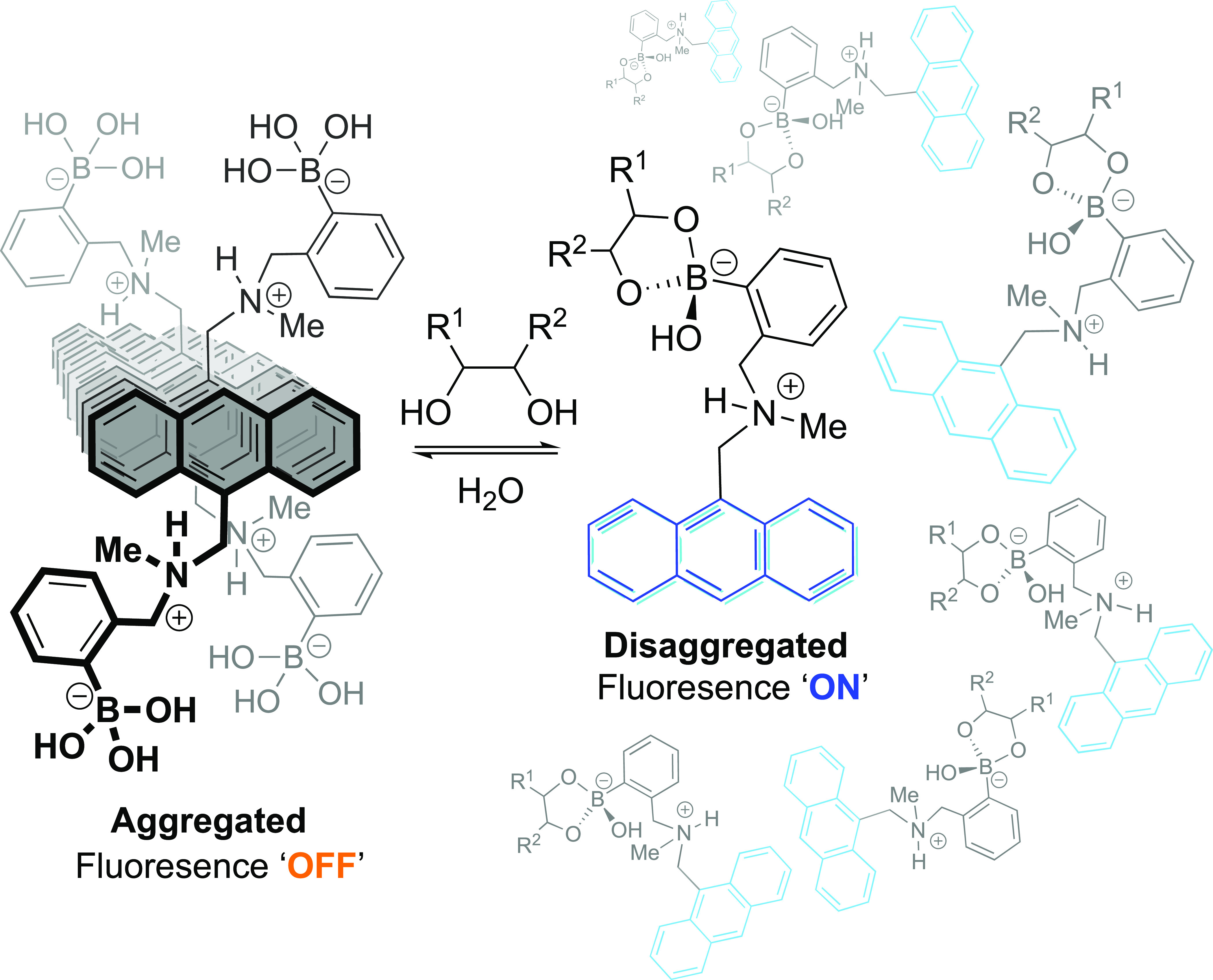
Fructose Binding Causing an Increase in the Solubility of the Sensor Resulting in Disaggregation and Subsequently Causing a Fluorescence Increase

However, evidence is growing that as a significant mechanism of fluorescence modulation in the saccharide sensing afforded by **10** is disaggregation, rather than any type of PET, Scheme 5. Anslyn and co-workers observed that in a 2:1 water/methanol solution containing sodium chloride (50 mM), boronic acid derivative **10** exists as a ground-state aggregate which forms (hitherto largely unreported) excimers upon irradiation.^76,87^ It was found that various stimuli resulted in disaggregation, including irradiation and sonication as well as fructose (**2**) addition; all producing the same increase in fluorescence emission (λ_em_ = 417 nm) and decrease in excimer emission (λ_em_ = 520 nm). Conversely, when using a purely methanolic solution, in which **10** is fully soluble, no aggregate-state excimer was observed, and the addition of fructose produced no change in fluorescence. While confirming that fructose certainly binds to compound **10**, the authors concluded that the diol–boronic acid-binding event, in itself, does not contribute greatly to the fluorescence switch-on of the sensor. Instead, it is predominantly the change in solvation properties engendered by the saccharide which indirectly leads to the emission turn-on; the solubility of **10** increases, resulting in disaggregation and a concomitant change of photochemical character.

**Figure 3:**
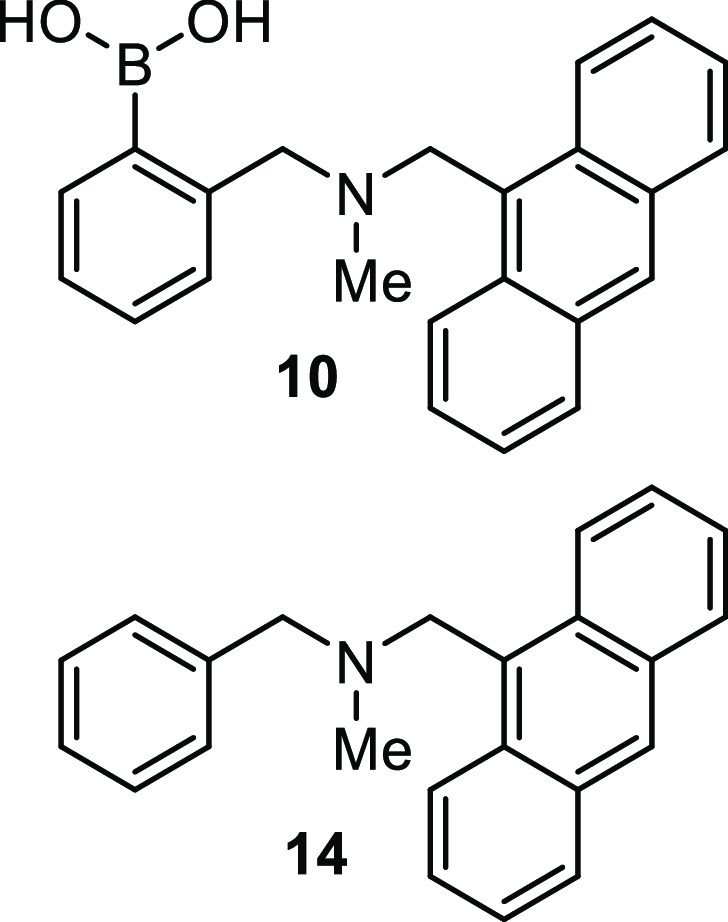
Upper: Structure of boronic acid-based saccharide sensor **10**. Lower: Control compound **14** used by Chaplin et al. to determine the influence of fructose-mediated disaggregation upon fluorescence.

Perhaps most importantly, Chapin et al. examined the fluorescence response of compound **14** to fructose (**2**). Compound **14** is the boron-free analogue of sensor **10**, Figure 3.^87^ Despite its lack of a covalent saccharide-binding moiety, it displays a clear fluorescence increase in response to fructose. Furthermore, the fluorescence data obtained by titrating fructose with this compound can be fitted to a one-to-one isotherm, with the same binding constant (within error) as that measured for compound **10**. Thus, Chaplin et al. concluded that while fructose undoubtedly binds to the boronic acid in **10**, it is not this that contributes the majority of the emission response but is, instead, the addition of fructose altering the solubility of the compound, thus causing disaggregation.^87^

These findings are, in hindsight not inconsistent, given that both the quenching of fluorescence and the formation of excimers (excited-state dimers) upon the aggregation of polyaromatic hydrocarbons such as anthracene and pyrene have been widely utilized in sensing systems for many years.^88−91^ Indeed, for some time boronic acid-based saccharide sensors have been designed to exploit this very phenomenon, often displaying enhanced selectivity.^92−95^

**Figure 4:**
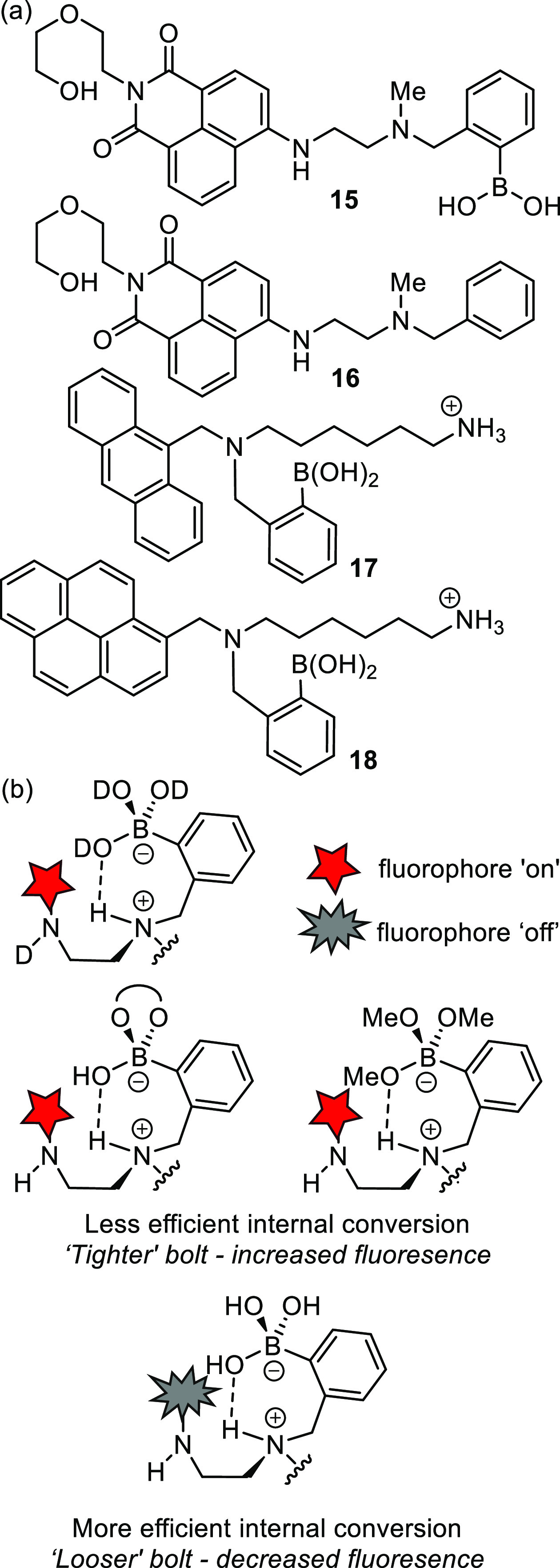
(a) Structures of the four compounds investigated by Sun et al. and (b) pictographic representation of the Sun et al. “loose-bolt” theory of fluorescence turn-on in response to saccharides.^96^

Subsequently, Sun et al.^96^ provided an insightful photochemical analysis, in which another subtle but important mechanism for emission turn-on is elucidated. Observing consistent behavior across four compounds **15**–**18**, as well as the classic sensor **10**, the findings are thought to be general, with relevance to all fluorescent boronic acid-based sensors for saccharides of this type (Figure 4a). Interestingly, following the addition of fructose, these compounds exhibit a significant fluorescence enhancement in water which was not observed in methanol. PET and p*K*_a_-switch effects were once again ruled out, with compounds **10** and **15** clearly exhibiting solvent insertion in protic media. Likewise, compounds **15**, **16**, and **17** were readily soluble in water, thus precluding any effects due to aggregation. To explain the findings, Sun et al. proposed a new “loose-bolt” internal conversion mechanism, in which the hydroxyl groups of the boronic acid act as an appropriate energy sink which quenches the fluorescence of the excited-state fluorophore, Figure 4b. Conversely, boronate esters (and even deuterated acids), as formed in the presence of methanol, saccharides, or D_2_O, have a greater mass, do not share the same vibrational states, and hence do not constitute an appropriate energy sink; fluorescence is thereby activated.

In an attempt to produce a unifying theory of the mechanism of fluorescence turn-on in sensors containing (*o*-(aminomethyl)phenyl)boronic acids, Sun, together with the groups of James, Wang, and Anslyn, published a treatise, outlining the role of the *o*-aminomethyl group.^97^ Data from each of these groups are presented, with a collegiate conclusion that the role of the *o*-aminomethyl group in the chemical structures of the discussed sensors is the lowering of p*K*_a_ of the boronic acid/ester group. The ammonium cation that forms upon solvent insertion is shown to catalyze the departure of leaving groups, owing to its acidic nature.^97^ Despite this change in the p*K*_a_, the collaborative team concludes that neither this PET nor a p*K*_a_-switch mechanism is operative, and that the evidence suggests that the *o*-aminomethyl group in fact has no role.^97^ The lack of a full and unified explanation of the mechanism of fluorescence turn-on in the classic Shinkai–James sensor has not prevented other researchers from developing a variety of sensors for carbohydrates with boronic receptors. As such, hereon in this review those recently developed sensing systems are presented, including both single-molecule and polymer-bound sensors, with optical, electrochemical, and NMR shift outputs.

**Figure 5:**
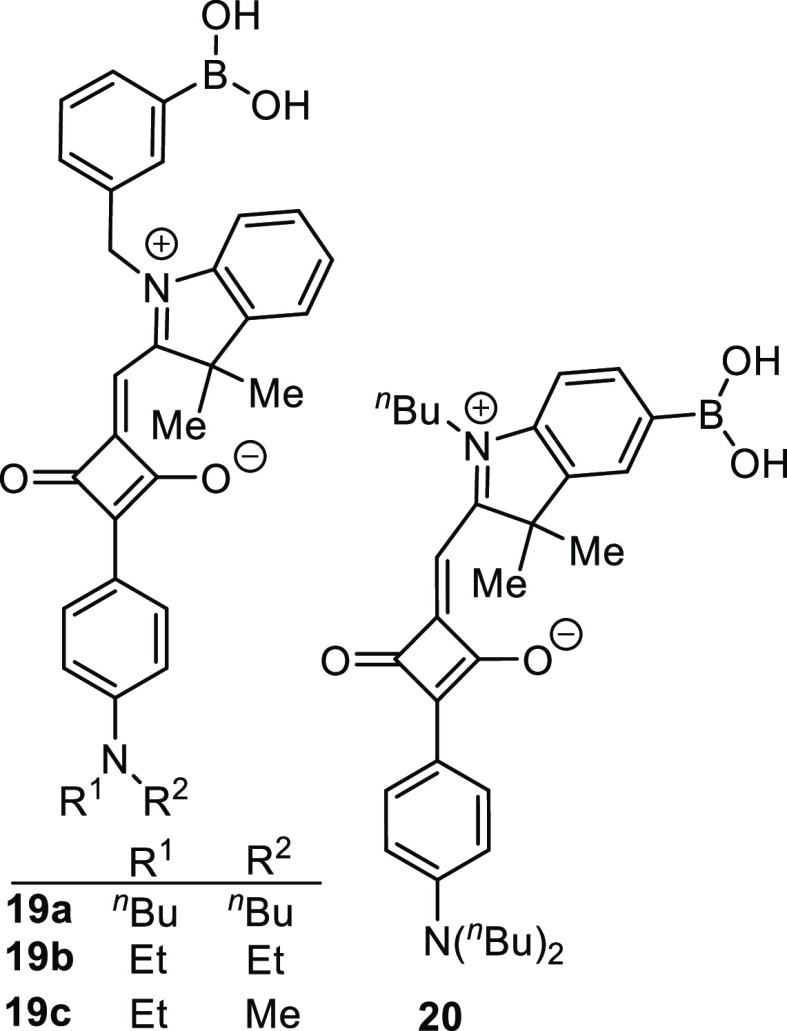
Squarilium cyanine-based fluorescent sensors for sialic acid.

Through the varied conjugation and incorporation of phenylboronic acid (PBA), Ouchi et al.^98^ developed four squarilium, cyanine-based fluorescent sensor molecules (Figure 5) which, depending upon the length of the alkyl side chain, **19a**–**c** and **20**, interact with sialic acid (Neu5Ac) to form structurally distinct aggregates with different emission profiles. Increased expression of sialic acids, which are an important biomarker used within cancer diagnosis, is associated with increased tumor growth and metathesis.^99−101^ Thermodynamic studies and structural analysis revealed that the shorter-chain-length dye formed a 2:1 J-aggregate with the saccharide, which was accompanied by a 2.4-fold increase in emission. Conversely the longer-chain-length dye formed a 1:1 H-aggregate with the saccharide, producing no fluorescence enhancement compared to the unbound sensor alone. The four sensor molecules were together employed in a multiple discriminant analysis assay (MDA) of human urine which provided successful discrimination of four clinically relevant concentrations (0.3, 2.0, 6.0, and 20.0 mM) of Neu5Ac, representing healthy to severely diseased states. While the presence of interferents such as proteins, peptides, and lipids meant the urine samples required filtration prior to analysis, which may constrain some practical applications, the results show great promise for such systems as molecular diagnostic tools. This is the first example of a Neu5Ac-selective chemosensor which is effective in purely aqueous media requiring no other cosolvents.

**Figure 6:**
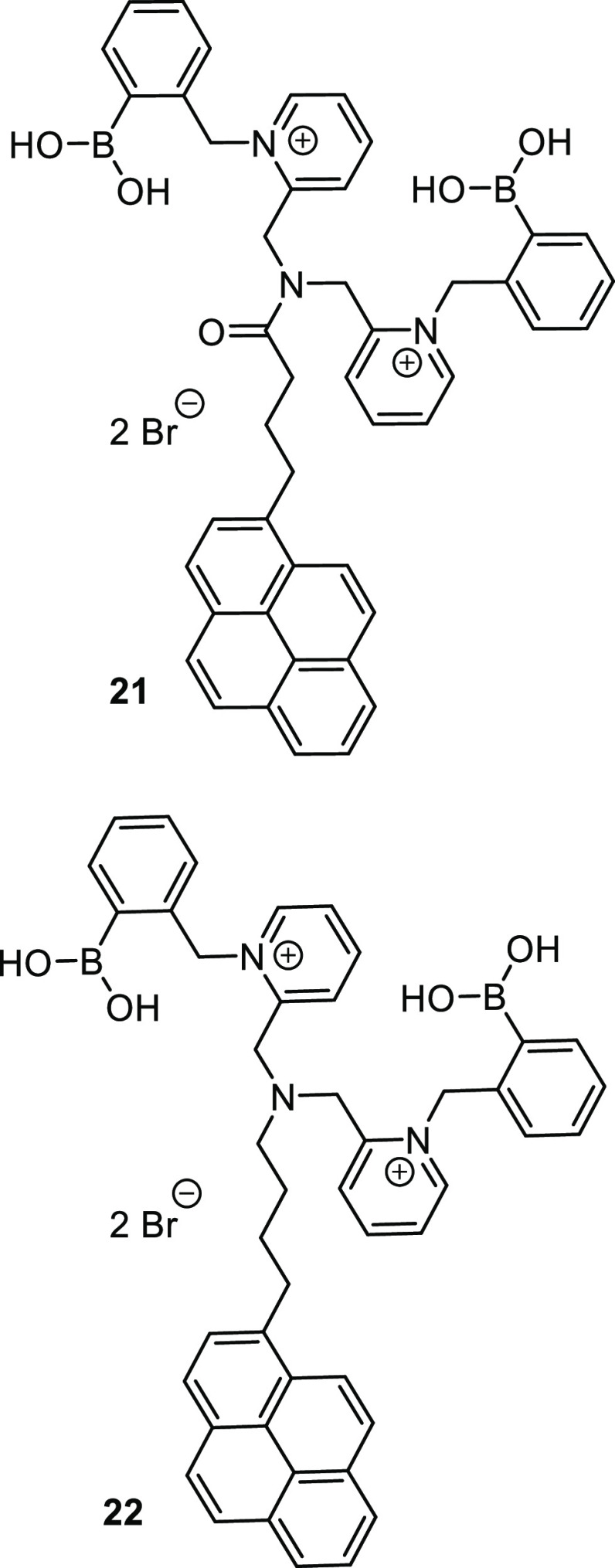
Two bispyridinium-based boronolectins synthesized by Zhang et al., **21** and **22**.^102^

Building on previous work with bispyridinium-based boronolectins,^102^ Zhang et al. synthesized two closely related water-soluble sensor molecules, in which a diboronic acid-binding site is connected via a flexible four-carbon linker and an amide or tertiary amine group, respectively, to a pyrene fluorophore. Although superficially similar, the two receptor molecules (**21** and **22**, Figure 6) interact quite differently with multivalent saccharide analytes to produce distinct assemblies. The sensor–saccharide complexes formed following a recognition event coalesce to form aggregates which produce distinctive excimer emission profiles.

While both monomers **21** and **22** produced peak emission at 381 nm, upon the formation of aggregates, distinct excimer emissions were produced at 528 and 510 nm, respectively. Upon the titration of different saccharides, the monomer emission of **21** decreased and the excimer emission increased in all of the cases except ribose, which exhibited no change. However, **22** produced a different set of results, while xylose and galactose (**3**) produced a decrease in the monomer emission, fructose and mannose produced an increase. For **22**, both ribose and glucose produced no change in the monomer fluorescence intensity. Both receptors produced increased excimer emission in the presence of all saccharides, except ribose which elicited no change in the emission of **21**. It was noted that the aggregates formed by the sensors could take some time to form; after combining sensor and analyte, the samples required aging for 3 h before the fluorescence profile stabilized. Alkaline conditions (pH = 10) were also necessary to prevent background excimer emission. Notwithstanding these practical constraints, by monitoring both monomer and excimer emissions, Zhang and co-workers used these two novel sensors to construct a four-channel assay, which, using linear discriminant analysis (LDA), could distinguish six monosaccharides at 100 μM concentration. This new assay enabled accurate sensing of glucose in artificial urine and blood containing common interferents, demonstrating the efficacy of the system under physiologically relevant conditions.

**Scheme 6:**
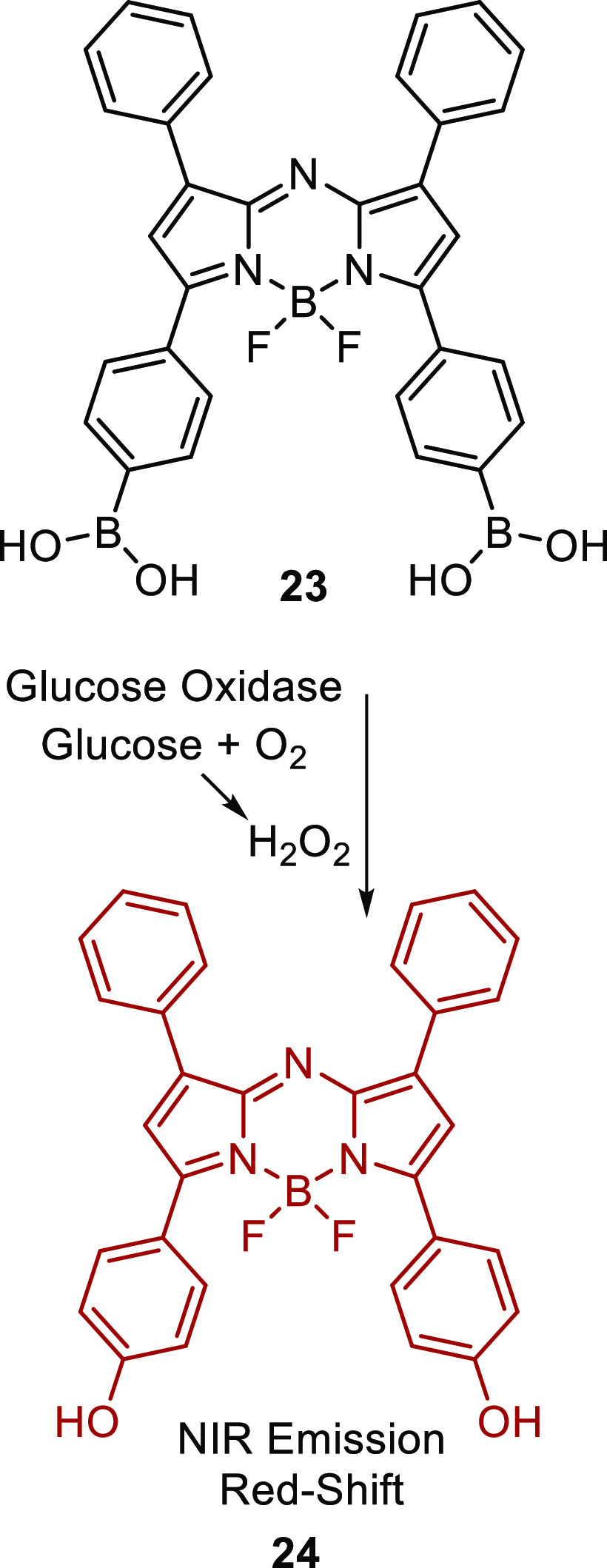
Glucose Oxidase-Mediated H_2_O_2_ Oxidation of Boronic Acid-Containing Chemodosimeter **23** Yielding **24**

To overcome some of the drawbacks such as cost and additional calibration associated with the previously described typical glucose oxidase based glucose monitors, Jiang et al. have developed a ratiometric fluorescent aza-bodipy-derived glucose dosimeter **23**.^103^ The same fundamental enzymatic process results in the quantitative oxidation of an electron-deficient PBA aza-bodipy to the corresponding phenol **24**, Scheme 6. This, in turn, gives rise to a detectable red shift in the NIR emission wavelength from 682 to 724 nm, resulting in a dual-wavelength sensor which benefits from minimal background interference. Although poor solubility required the use of 50% ethanolic buffer, the dosimeter was successfully enmeshed within a polymer film to produce a glucose optode, which was used to quantify glucose from 60 μM to 100 mM in 40-fold diluted whole blood. Despite its sensitivity, even within whole blood, the oxidation of the boronic acid renders this system unsuitable for continuous glucose monitoring applications, owing to its consumption of the reporter.^103^

**Figure 7:**
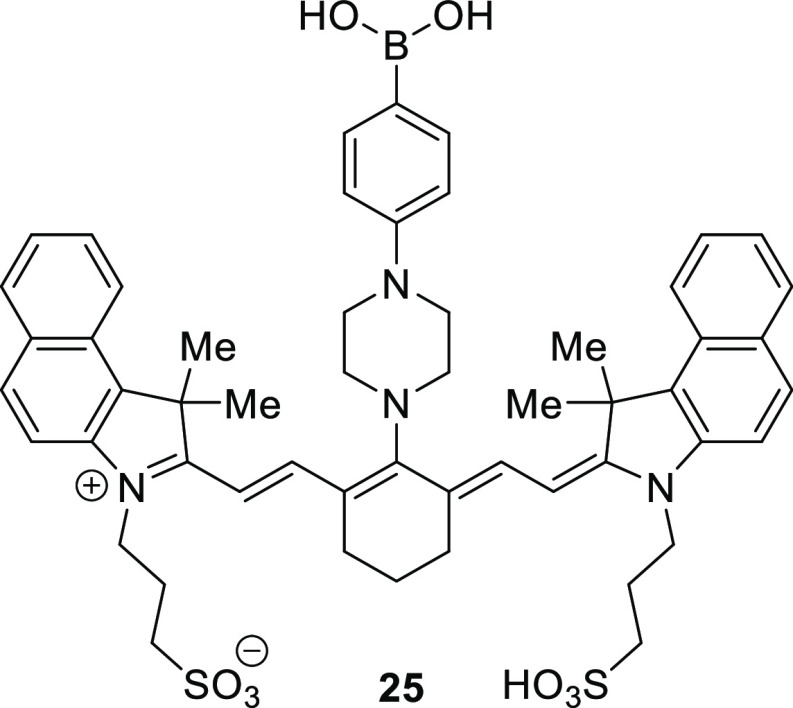
Long wavelength, tricarbocyanine-derived glycosylated mucin sensor **25** produced by Lopez et al.^104^

Exploiting the low NIR autofluorescence of biological systems, Lopez et al. developed a novel tricarbocyanine-derived boronic acid-based molecular sensor suitable for both in vitro and live cell labeling, **25**, Figure 7.^104^ Unlike many synthetic boronolectins, this probe is readily water-soluble owing to its zwitterionic nature and displays an acceptable off–on response at physiological pH; no organic cosolvents were required. With an emission wavelength of 820 nm at pH 7.4, this probe offers the longest wavelength probe at physiological pH that had been reported at the time of publication. The greatest fluorescence turn-on response was achieved in the presence of sorbitol and fructose, with binding constants calculated to be 3.3 and 1.5 M^–1^, respectively. Promising glycoprotein responses were also demonstrated; the system was particularly sensitive to the presence of mucin owing to its high glycosylation level and produced a detectable change of fluorescence intensity at concentrations as low as 300 nM. Suitability of this boronolectin for imaging applications was found using it with confocal microscopy of MCF-10 cells. Probe **25** was shown to illuminate the endoplasmic reticulum and the Golgi apparatus, two organelles in which the glycosylation of proteins is known to occur,^105^ indicating its suitability for such purposes.

**Scheme 7:**
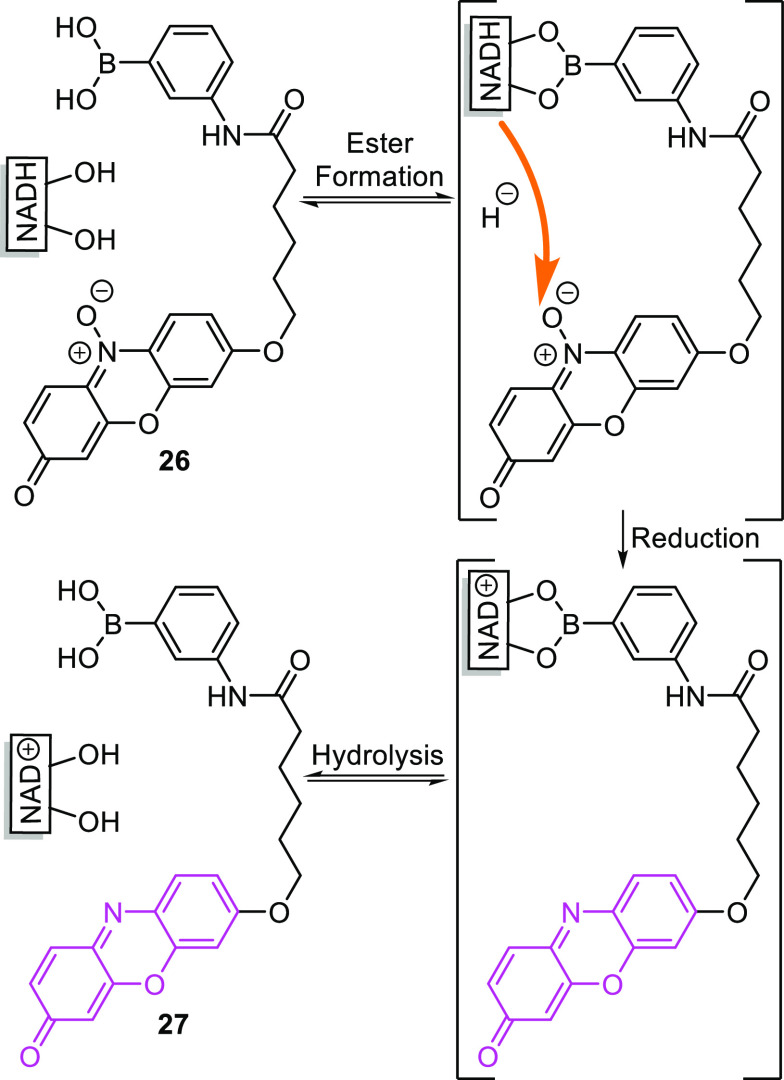
Reduction of Senor **26** by NADH to Become Fluorescent **27** As Reported by Wang et al.^107^

Inspired by the enzymatic cycling assay used for *in vitro* NADH quantification since the 1960s,^106^ Wang et al. developed a strategy that consists of a similar two-step process.^107^ First, the reporter molecule **26** binds to NADH through the interaction between boronic acid and the ribose saccharide unit. Now, in close proximity, a hydride transfer from NADH reduces the weakly fluorescent resazurin reporter group to the strongly fluorescent resorufin (**27**), Scheme 7. Using a simple boronic acid as the binding site, the system displayed excellent sensitivity with a detection limit of 0.087 μM, although it required a buffered DMSO/water mixture at pH 9.5 for optimal operation. In an attempt to increase selectivity, a second-generation probe was developed, in which the boronic acid was converted to a benzoxaborole. This improved the performance of the probe, which displayed enhanced selectivity in the presence of common biological interferents and a detection limit of 0.41 μM under physiologically relevant (pH = 7.4) conditions in the absence of enzymes. The probe was shown to have low cytotoxicity and was capable of imaging and quantifying NADH within live oral squamous cell carcinoma cells.^107^

**Figure 8:**
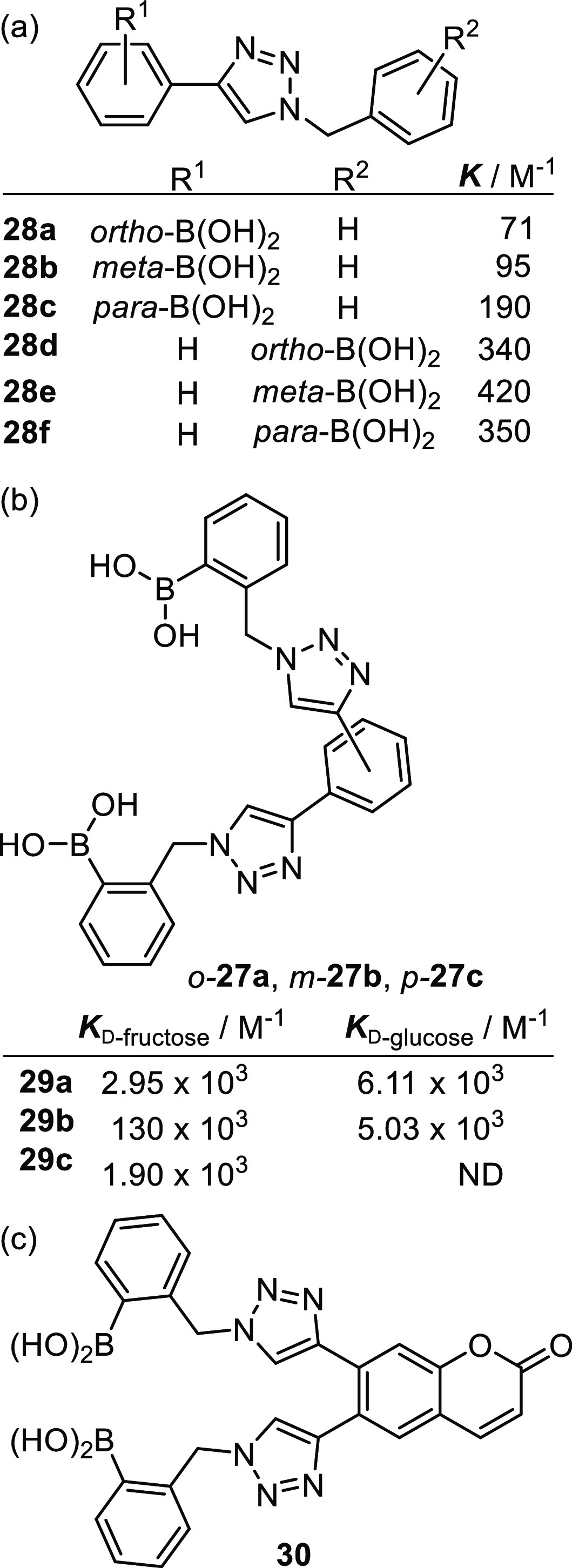
Fossey and co-workers’ development of saccharide-sensing *Click-fluors*.

Previous work by Scrafton et al.^108^ and Zhai et al.^109,110^ has demonstrated that so-called *click* chemistry can be successfully employed in the synthesis of modular, fluorescent boronic acids (work coauthored by one of the authors of this review). It is hoped that demonstration of boronic acid derivatives’ amenability to such a modular approach will allow rapid access to relatively large libraries of potential saccharide sensors. The group employed copper(I)-catalyzed cycloaddition conditions developed by Molander and Ham^111^ to couple phenylboronic ester derivatives producing a fluorophore via installation of a triazole linkage, thereby producing an example of so-coined *click-fluors*,^108^Figure 8a, **28d**. It is conceded by the authors who coined the term click-fluor (who include one coauthor of this review) that the reaction conditions used and outcomes observed fail to meet Sharpless’ definition of “click chemistry”.^112,113^ Although lower catalyst loadings helped to prevent copper-catalyzed deborylation, purification of product from unwanted byproducts was still required. This methodology has subsequently been employed to produce the six regioisomers, **28a**–**f**, allowing the binding characteristics to be interrogated and correlated to structure (relative spatial arrangement of boronic acid motifs). Isothermal titration calorimetry (ITC) experiments revealed that all cases, triazoles with the boronic acid unit tethered at the 1-triazole position (**28d**–**f** derived from the azido-boronic ester) produced higher binding constants with d-fructose than those with the boronic acid unit at the 4-triazole position (**28a**-**c** derived from the alkyne-boronic ester) in methanolic buffer, pH 8.21. It is proposed that the latter are deactivated due to π-conjugation with the electron-rich triazole, rendering the boron less electrophilic/Lewis acidic. Under these conditions little difference in binding strength is observed between the ortho-, meta-, and para-forms; however, a further ^1^H NMR spectroscopy titration study suggested that the ortho-isomer binds d-fructose more strongly than the para-isomer. A further study of eight compounds bearing triphenylamine, coumarin, 1,8-napthalimide, and pyrene fluorophores at the 4-position confirmed that fructose-binding fluorescence enhancement was also most pronounced when the boronic acid is ortho to the triazole.

In 2017 Fossey and co-workers extended the scope of this methodology to produce a series of bis-boronic acid click-fluors based on the most promising, previously established binding motif, with the intention of developing multivalent receptors for glucose.^114^ Hence, two *o*-azido-boronic esters were connected via each of the bisalkynes: 1,2-, 1,3-, and 1,4-phenylene bis-alkynes to produce three regioisomeric structures (Figure 8b), **29a**–**c**, and their saccharide binding was studied by ITC. The strength of glucose binding increases across the para, meta, and ortho series, indicating that appropriate spacing between the boronic acid groups is crucial for selectivity. Indeed, producing a binding constant twice that observed with fructose, the ortho-isomer is somewhat glucose selective. Conversely the meta-isomer exhibits exceptional d-fructose selectivity, producing a binding constant twenty-six times that of d-glucose (PBS buffer with 20% DMSO, pH = 8.21).

Having identified the most promising glucose-selective regioisomer, the motif was modified to incorporate a coumarin fluorophore producing sensor-molecule **30**. Interestingly, the fluorescence of sensor **30** is enhanced in the presence of fructose but decreased in the presence of glucose. In both cases a saccharide concentration of 1.0 mM produced a detectable change in fluorescence intensity (methanolic buffer, pH = 8.21). The authors conclude that this must be due to the different binding modes of each sugar; crystal structures of the corresponding pinacol boronic esters (boronic ester of **29a**–**c**) suggest torsional constraints imposed by the ortho-substituted phenylene linker of **29a**-type glucose-selective motif may be responsible for both appropriate positioning of the boron atoms in the binding domain and the observed impact upon fluorescence response of **30**; full elucidation of such mechanisms remains an ongoing endeavor.

**Figure 9:**
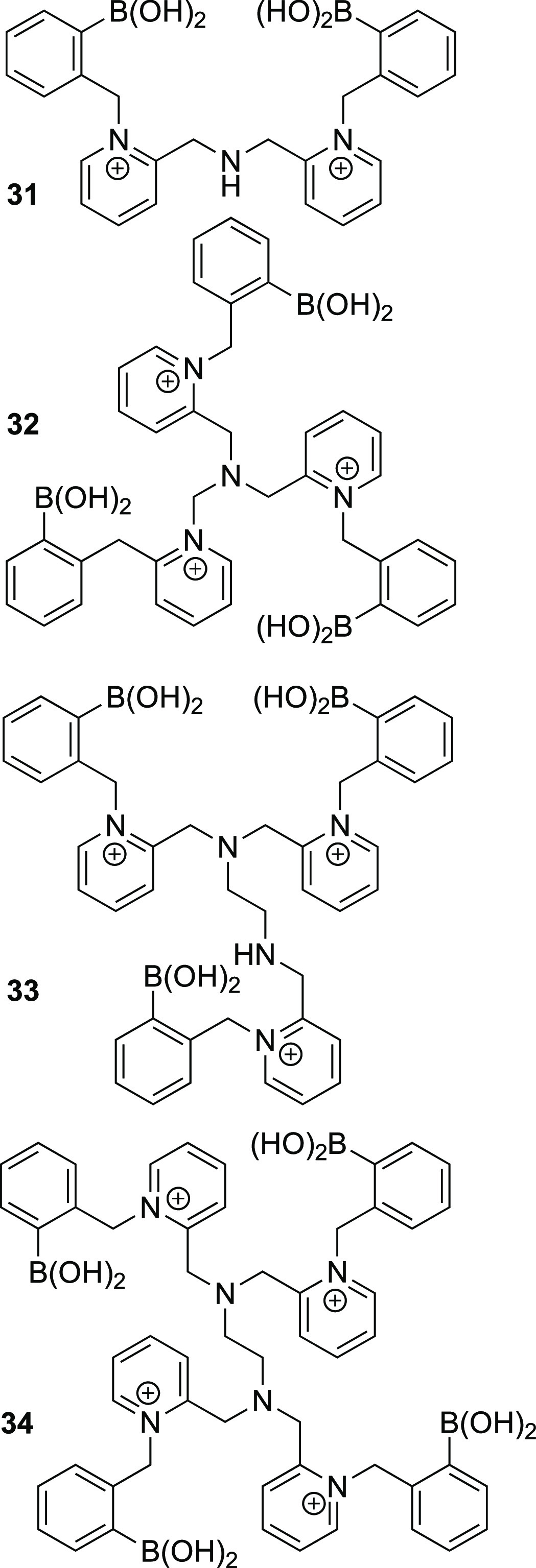
Four boronolectins that make up the “synthetic tongue” as described by Zhang et al.^117^

Zhang employed boronic acids in the development of an “artificial tongue” which the authors claim is suitable for high-throughput sensing of ginsenoside glycoconjugates. The sensing mechanism is based upon the (previously reported) superquenching effect of cationic pyridinium salts upon otherwise fluorescent, anionic poly(phenylene-ethynylene) (PPE) electrolytes.^115^ In the absence of analyte, the strong electrostatic force of attraction facilitates rapid electron transfer between the two oppositely charged components, resulting in quenched fluorescence. Upon binding a diol-bearing analyte, the neutral boronic acid units are transformed into negatively charged boronate esters, canceling out the positive charge of proximal pyridinium ions through the formation of zwitterions. This overall reduction in positive charge lessens the association between receptor and polyelectrolyte, thus restoring fluorescence.^116^ Four multivalent boronolectin molecules bearing two, three, or four pyridinium-linked PBA-binding sites were synthesized, **31**–**34**, Figure 9.^117^ Each of these flexible, cationic, carbohydrate-binding molecules were then incorporated within two fluorescent, anionic polyelectrolyte systems to produce eight distinct sensing channels. Each channel exploits two possible opportunities for discrimination; the first is the idiosyncratic interaction between receptor and analyte; the second is the unique relationship each resultant host–guest complex has to the polyelectrolyte. Using LDA, this eight-channel array correctly distinguished 13 out of 15 samples, composed of five different ginsenosides at three different concentrations with 86.7% accuracy.^117^

**Scheme 8:**
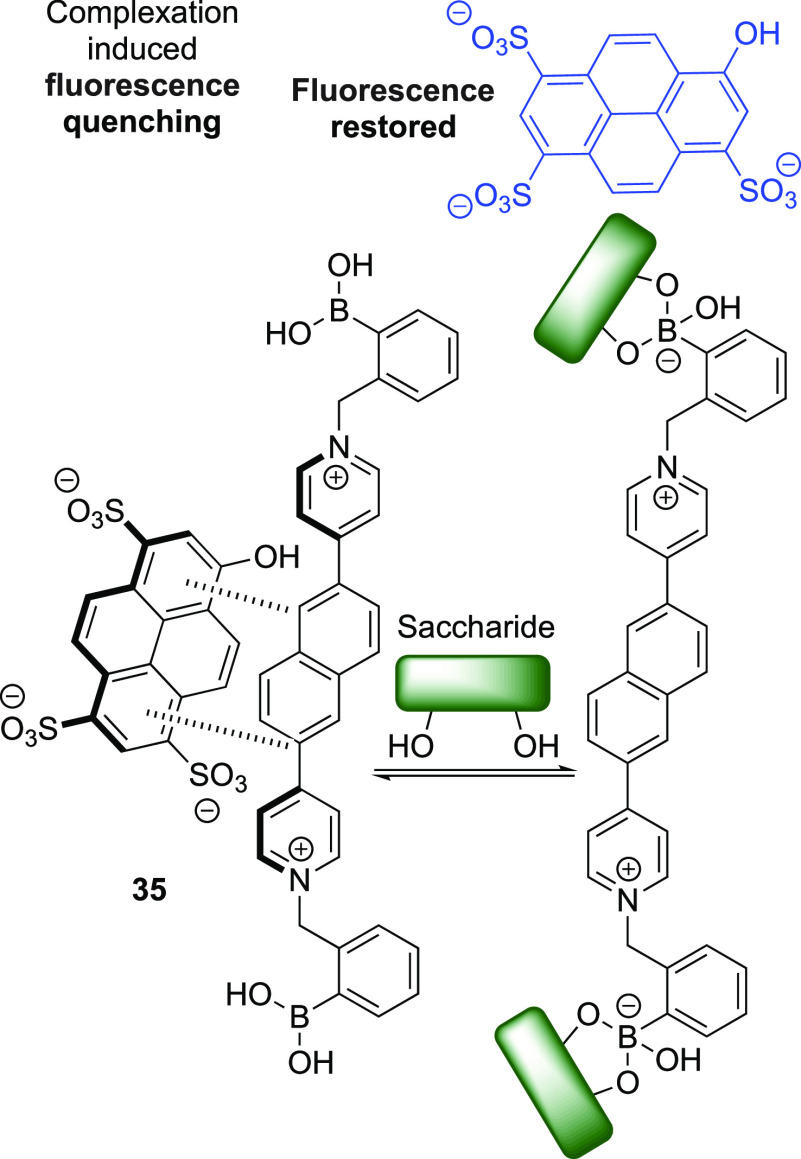
Ability of Sensor **35** to Act as an IDA for Saccharide Detection

Combining a naphthylpyridinium core with the affinity of boronic acids for diols, Resendez et al. developed sensor **35**, capable of acting as both a two-component indicator displacement assay (IDA), Scheme 8, and as a single-component ICT chemosensor (not shown).^118^ IDAs are a subset of chemosensors that use competitive binding of a reporter and an analyte to a receptor to determine analyte concentration. For a recent outline of advances in IDA development, readers are directed to a review by Sedgwick et al.^119^ The cationic pyridinium moieties electrostatically bind anionic dyes, such as 8-hydroxypyrene, 1,3,6-trisulfonic acid trisodium salt (HTPS), and tetrakis(4-sulfophenyl)porphine (TSSP), in turn quenching their fluorescence. Upon binding a saccharide, the p*K*_a_ of the boronic acid is lowered, which facilitates the formation of the anionic boronate ester. With the introduction of these anions, the cationic character of the molecule is neutralized, thus liberating the bound dyes, allowing their detection. There is a similar reliance on the boronic acid p*K*_a_ change in the single-component ICT system. With the formation of the boronate ester, and the subsequent sp^2^ to sp^3^ hybridization change, the ICT process is prevented, leading to a saccharide concentration dependent fluorescence increase.^118^

**Figure 10:**
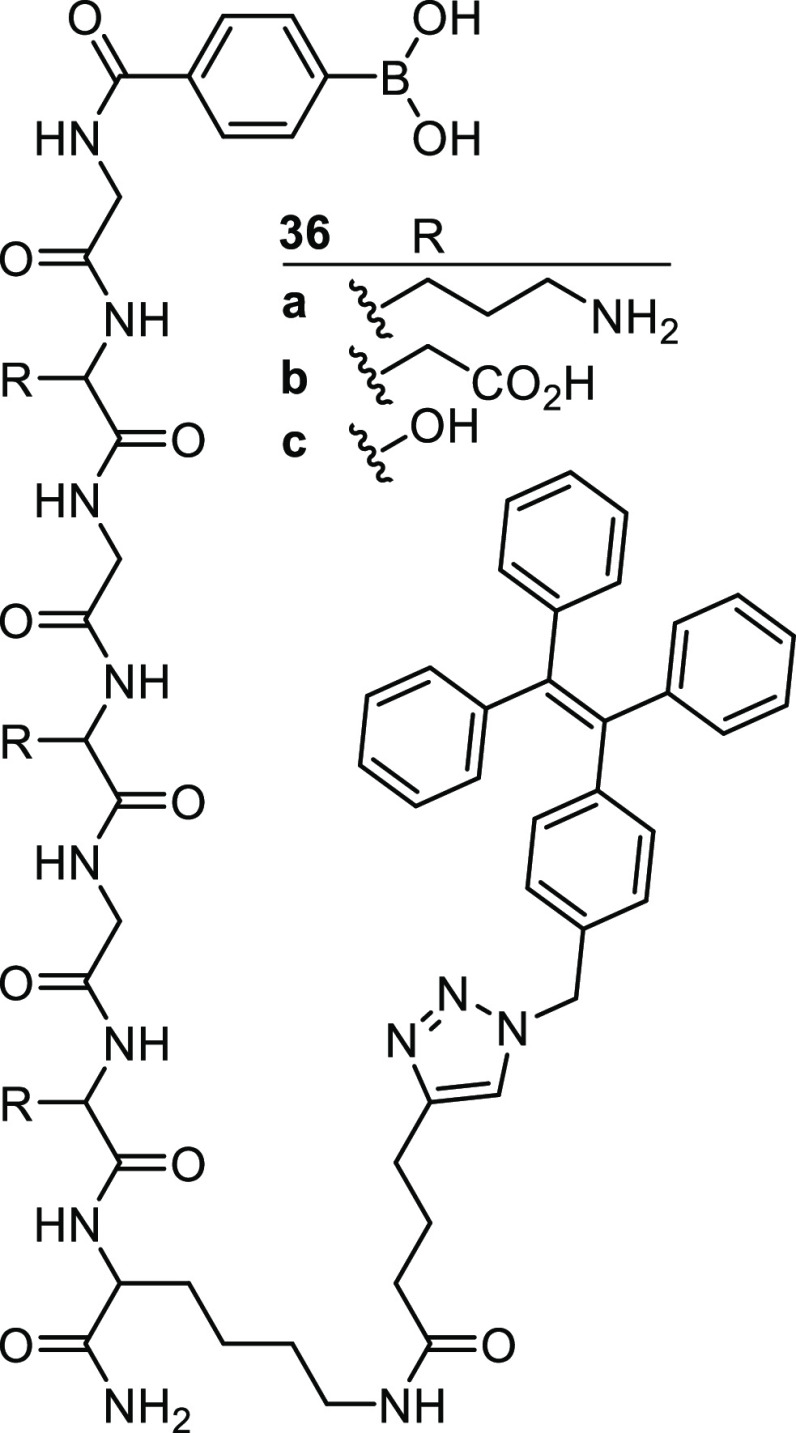
Sialic acid sensors **36a**–**c** consisting of a boronic acid-binding moiety bound to a TPE reporter through a modified GKGKGKK chain (G = glycine; K= lysine).

As previously discussed, the diol recognition ability of boronic acids enables their use in detecting carbohydrate-derived cancer biomarkers. Sialic acid is overexpressed in a variety of cancers, and as such Peng et al. produced a peptidic boronic acid tagged probe, capable of detecting this analyte on the surface of cancer cells.^120^ They report three sensors **36a**–**c**, which feature a boronic acid linked to tetraphenylethene (TPE) via a peptide chain composed of alternating glycine (G) and lysine (K) units, Figure 10. In its fully solvated form, TPE is nonfluorescent, however upon aggregation exhibits a fluorescence turn-on, a phenomena known as aggregation induced emission (AIE).^121^ The peptidic chains imbue the sensors with water solubility, as well as specificity toward sialic acid. The strongest binding and most selective of the sensors tested was **36a**, which exhibited a *K*_a_ of ∼327.8 M^–1^ toward sialic acid. It was hypothesized that these sensors would bind to sialic acid on the cell surface, enabling TPE aggregation with adjacent sensors which were bound to different molecules of sialic acid. This was proven using imaging experiments; human heptatic cancer (HepG2) cells are known to overexpress sialic acid, while AML-12 cells express very little. The observed fluorescence increases were in line with the authors’ predictions, and only the HepG2 cells triggered a fluorescence response. This approach circumvents the issues often present in other cancer diagnostic systems which include high costs and poor stability.^103,122^

**Figure 11:**
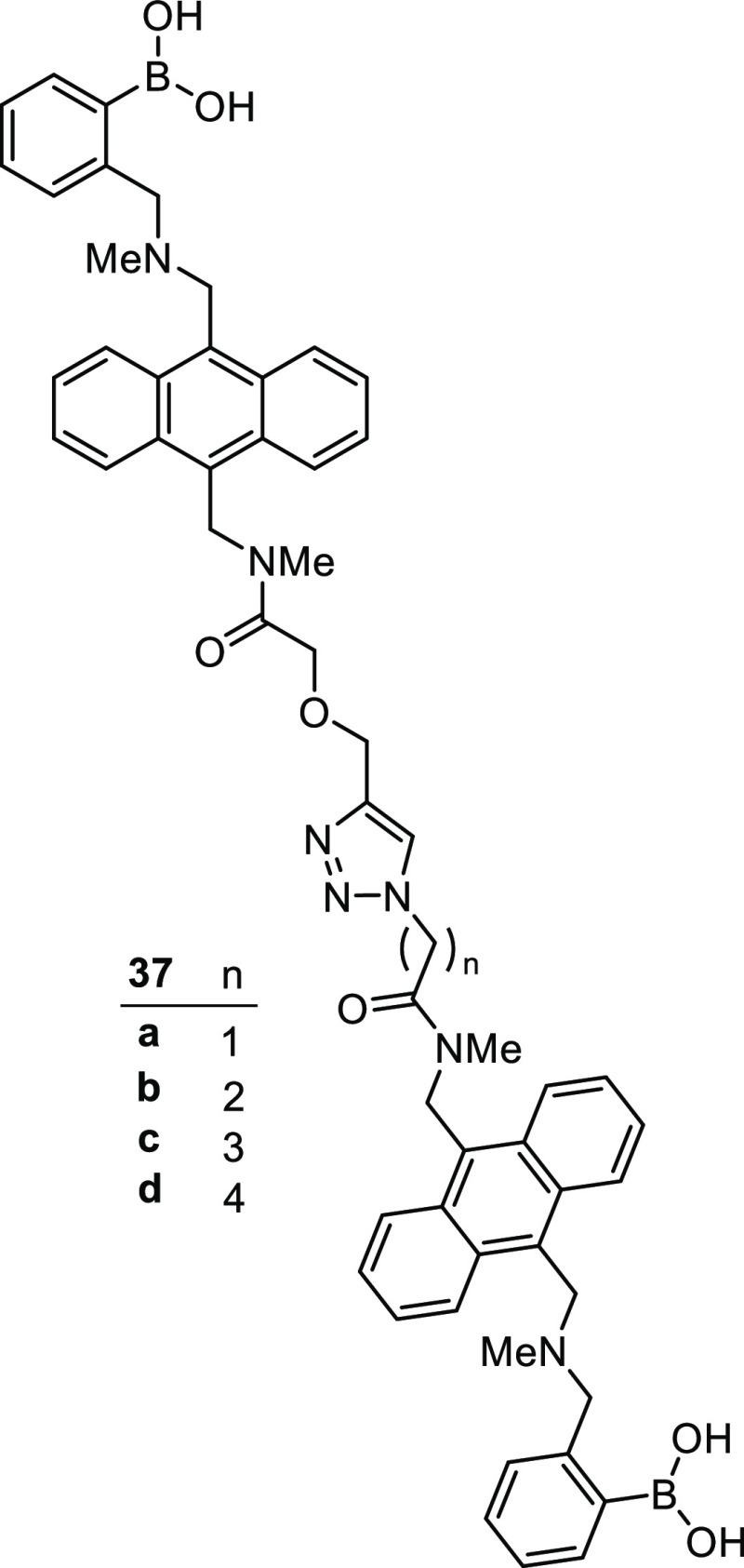
Triazole-linked sensors for the detection of Le^*y*^ as developed by Wang et al.^123^ Note the expected 1,4-triazole substitution pattern is drawn here, in contrast to the 1,5-pattern drawn by the authors of the original report.

Also harnessing the power of click chemistry, in 2017 Wang et al. produced a series of four fluorescent bis-boronic acids targeting the Lewis group of cell-surface tetrasaccharides, **37a**–**d**, Figure 11.^123^ In essence, two of the Shinkai and co-workers’ PBA-anthracene-type systems are appended with different linker lengths and are “clicked” together about a triazole core. Thus, the resulting bis-boronic acid, fluorescent receptors, all bear a large “bite”, suitable for spanning across the intended tetrasaccharide targets. Sensor **34c** displayed particular sensitivity, the presence of Lewis *y* (Le^*y*^) producing an enhancement of the fluorescent intensity of over 70%. One drawback of these sensors is the requirement for buffers with methanol contents of up to 60%, which makes them unsuitable for many biological purposes.

Sensor **37a**, although not displaying the greatest fluorescence enhancement, was the most selective for Le^*y*^. This selectivity for Le^*y*^ was demonstrated through cell-labeling experiments using laser scanning confocal microscopy. Sensor **37a** was shown to bind to HEP3B cells, which express only Le^*y*^, producing a strong signal under laser scanning confocal microscopy. Neither HEPG2 cells, which express only sLe^x^, or GES-1 cells, which do not express either antigen, were labeled, even at much higher concentrations of **37a**. Seeking to rationalize the selectivities, Wang and co-workers performed computational simulations of the sensor molecules to find the average distance between each boronic acid unit. While these preliminary results offer some insight into the potential conformations of compounds **37a**–**d**, the complexity of the binding modes indicates that further, more rigorous, studies would be required to draw meaningful conclusions.

**Figure 12:**
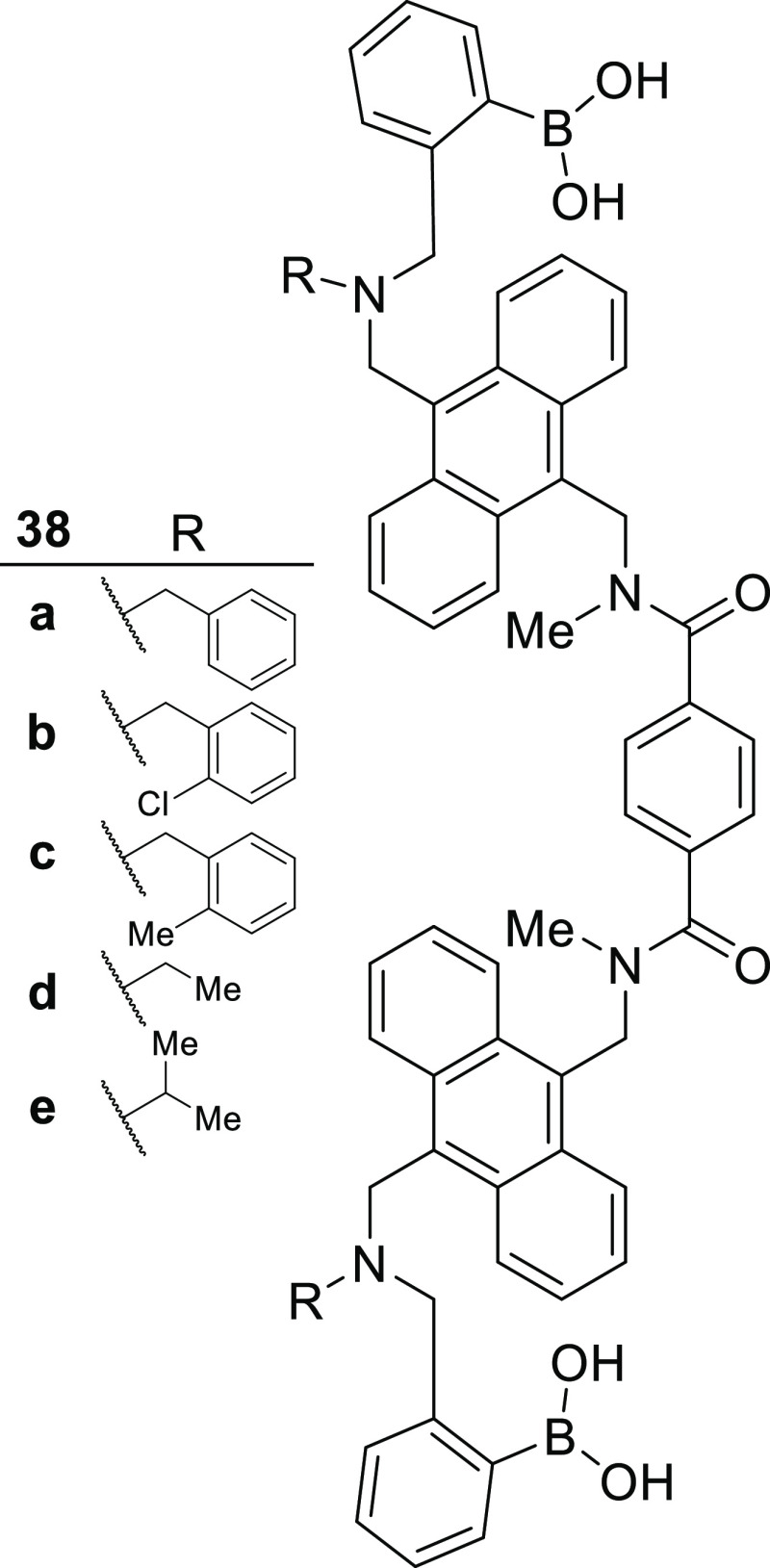
sLe^*x*^ sensors developed by Wang et al.^124^

Toward targeting a different oligosaccharide-based antigen, Li and co-workers developed an anthracene-based sensor for sialyl Lewis *x* (sLe^*x*^).^124^ The influence of steric bulk at the amine substituent adjacent to the anthracene was hypothesized to provide a level of selectivity toward specific oligosaccharides, through restriction of molecular orientation. Compounds **38a**–**e** were synthesized Figure 12, and their fluorescence intensities in the presence of Lewis acids *x* (Le^*x*^) and *y*, and their sialyl derivatives (sLe^*x*^ and sLe^*y*^) were measured at concentrations of 5 μM sensor and 60 μM analyte. It was found that compound **38a** offered the greatest selectivity toward sLe^*x*^ producing with a 4-fold increase in fluorescence when compared to the Le^*y*^ which produced the second highest signal. Compound **38e** produced a fluorescence turn-on signal in response to each of the tested oligosaccharides with little selectivity, however compounds **38b**–**d** produced only a weak response. Because all of the bis-boronic acids feature the same linker, it can be concluded that the differences in selectivity and sensitivity are due to steric contributions of the R group. Using MTT assays, each of these compounds was shown to be nontoxic at concentrations up to 20 μM, allowing their use in live cell imaging. It was shown that compound **38a** was capable of selectively imaging of HEPG2 cells that produce only sLe^*x*^, while compound **38e** also stained cells that expressed Le^*y*^.

Many researchers have sought to develop macromolecular boronolectins; these polymeric sensing systems often take the form of hydrogels, which offer advantages toward biological applications including flexibility, high biocompatibility, and tailorable mechanical properties.^119^ The immobilized nature of the receptor moiety (the boronic acid) lends itself toward continuous monitoring, while the highly solvated nature of the material allows for free diffusion of the analyte (saccharides). This is also a convenient strategy toward overcoming the solubility limitations that are inherent in many boronolectins.

**Scheme 9:**
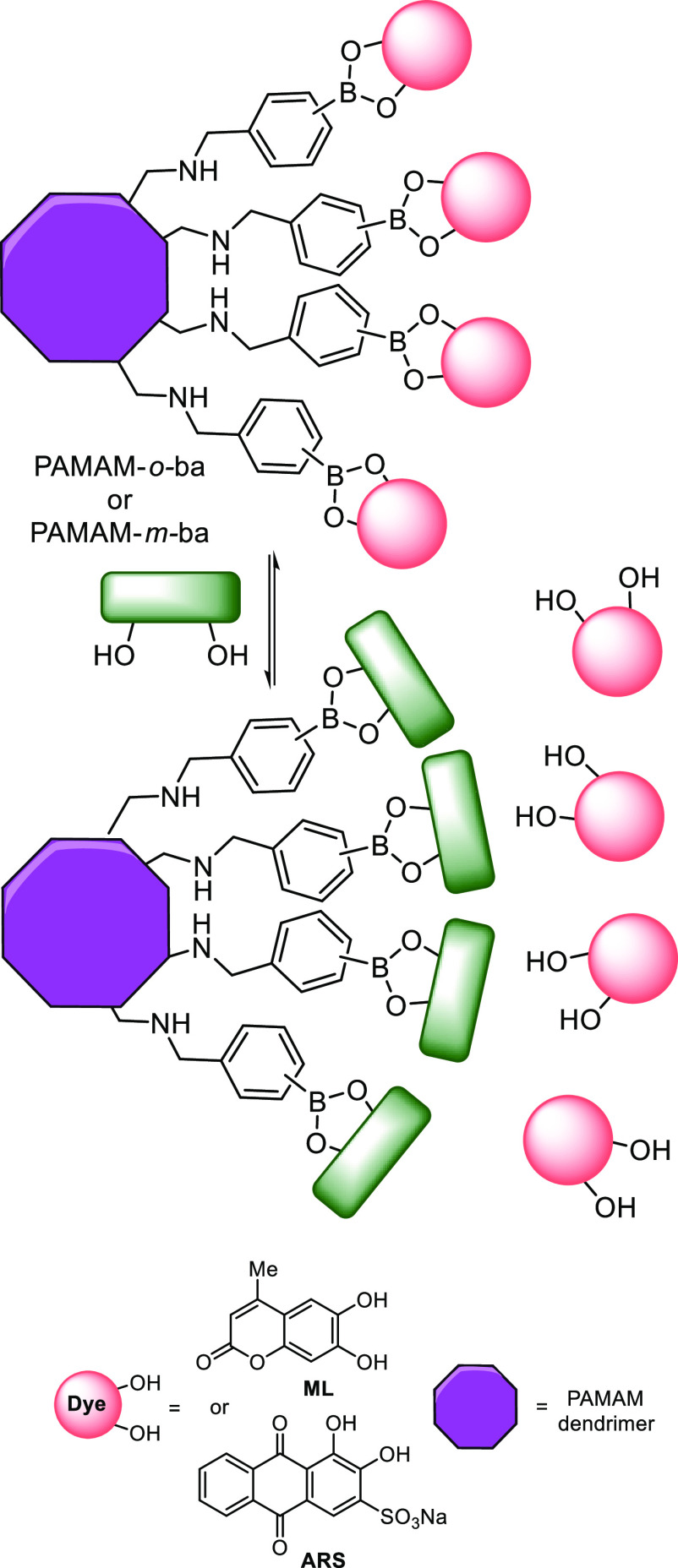
PAMAM–Boronic Ester–Dye Complexes Forming an IDA to Detect Saccharides

Liang et al. developed an IDA through the functionalization of poly(amidoamine) dendrimers with boronic acids, capable of detecting saccharides in water.^125^ These dendrimers, bound with *m*-boronic acids (PAMAM-*m*-ba) and *o*-boronic acids (PAMAM-*o*-ba), were loaded with two commercially available catechol containing dyes, alizarin red S (ARS) and 4-methylesculetin (ML), Scheme 9. Upon PAMAM–dye complexation, the fluorescence of the ML and ARS dyes are enhanced, and shifts in their absorbance are observed.^125^ The introduction of saccharides introduces an equilibrium between PAMAM–dye complexes and PAMAM–saccharide complexes, releasing either the ARS or the ML, producing a measurable fluorescence signal. While the system could detect the presence of saccharides at physiological pH (7.4), its sensitivity was greatly enhanced at pH 10.0. These dye-bound systems were tested for their ability to detect ribose, galactose (**3**), fructose (**2**), and glucose (**1**) using both fluorescence intensity and absorption. The ML loaded dye was also measured by monitoring fluorescence anisotropy; however, this was not possible with ARS due to its tendency to aggregate. Interestingly, it was found that each boronic acid dye complex interacted with each sugar in a unique response pattern. While the PAMAM-*m*-BA-ML complex was unable to distinguish between fructose and glucose, it was shown that the PAMAM-*m*-BA-ARS complex could; however, it in turn was unable to distinguish ribose and galactose. To overcome this, the authors designed a plate reader-based sensing array. Through LDA analysis, this high-throughput system was shown to be capable of discriminating fructose (**2**), glucose (**1**), ribose, and galactose (**3**).

**Scheme 10:**
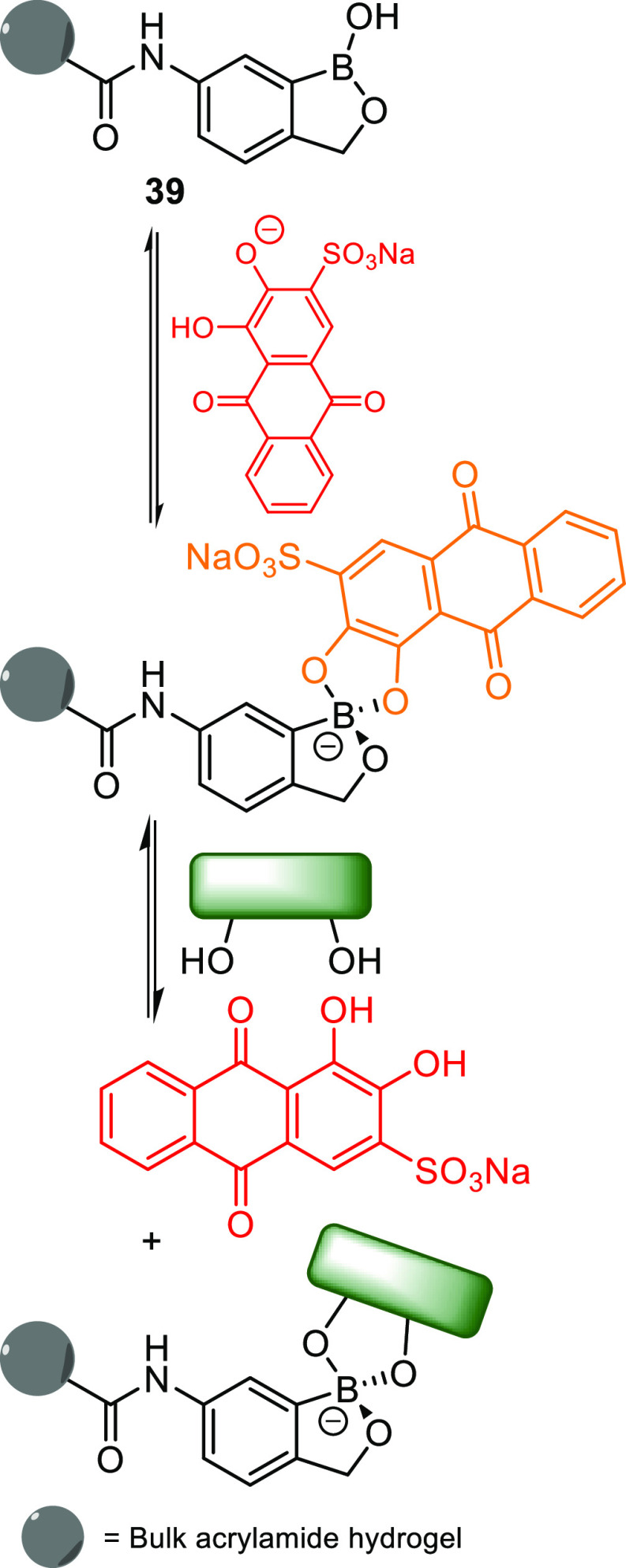
IDA for the Detection of Sugars Based on a Benzoxaborole-Containing Hydrogel

Ma et al. had previously synthesized a variety of boronic acid-containing acrylamide monomers which were subsequently polymerized into polyacrylamide hydrogels. These were loaded with ARS to form an IDA, which presented a colorimetric response on exposure monosaccharides, with increasing affinity to each sugar, in line with previously developed boron acid saccharide sensors.^126^ Building on this, Lampard et al. (together with one of the coauthors of this review) explored the use of benzoxaborole (BOB)-containing acrylamide monomers, **39** (Scheme 10).^127^ Benzoxaboroles have been known to exhibit a greater affinity toward saccharides^128^ and, as such, were compared to the previously reported boronic pinacol ester gels. The benzoxaborole-containing gels offered a greater binding affinity toward each of the monosaccharides tested, rather than the pinacol ester, as determined by the release of ARS which was measured as a function of absorbance increased at 513 nm. The BOB gels were capable of increasing the dye release in response to the saccharide by 14% with fructose (**2**), 30% with galactose (**3**), 43% with mannose, and 56% in response to glucose (**1**).^127^

**Figure 13:**
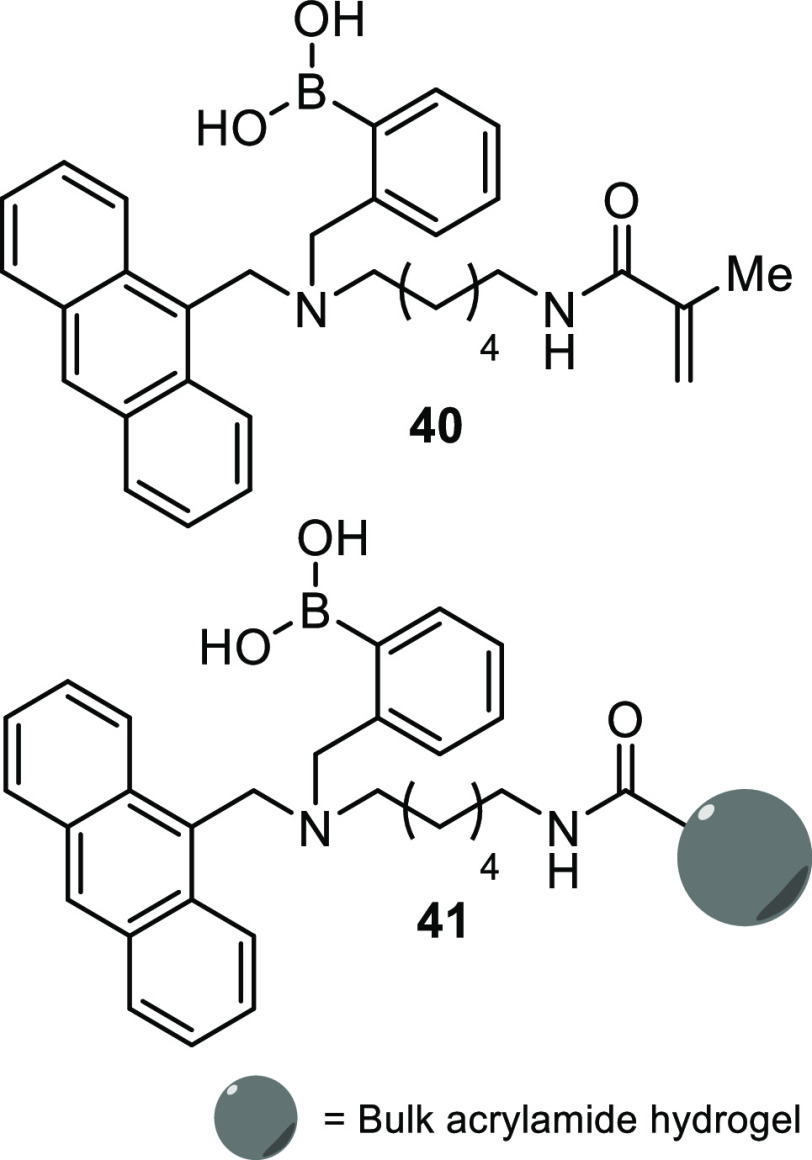
Anthracene boronic acid acrylamide monomers and hydrogel as developed by Xu et al.^129^ for detecting saccharides.

Further research into the use of boronic acid-containing acrylamide monomers for the formation of saccharide-sensing hydrogels has been conducted by Xu et al., together with two coauthors of this review.^129^ As opposed to previous efforts which utilized ARS in the development of a polyacrylamide immobilized IDA, a polyacrylamide hydrogel capable of directly binding to saccharides with a fluorescent output was developed.^129^ To achieve this, an (*o-*aminophenyl)boronic acid, with an anthracene reporter, was linked via a six-carbon chain to an acrylamide unit, **40**, Figure 13. This was then copolymerized with acrylamide to form a polyacrylamide hydrogel, yielding material **41**, Figure 13. The fluorescence enhancements of both the free monomer and the hydrogel were measured in the presence of four monosaccharides: d-glucose, d-fructose, d-mannose, and d-galactose. As is to be expected, the increases in fluorescence were observed in the order d-fructose > d-galactose > d-mannose and d-glucose; however, there was a notable decrease in sensitivity when the sensor was immobilized into the hydrogel when compared to the solution-based experiments.

**Figure 14:**
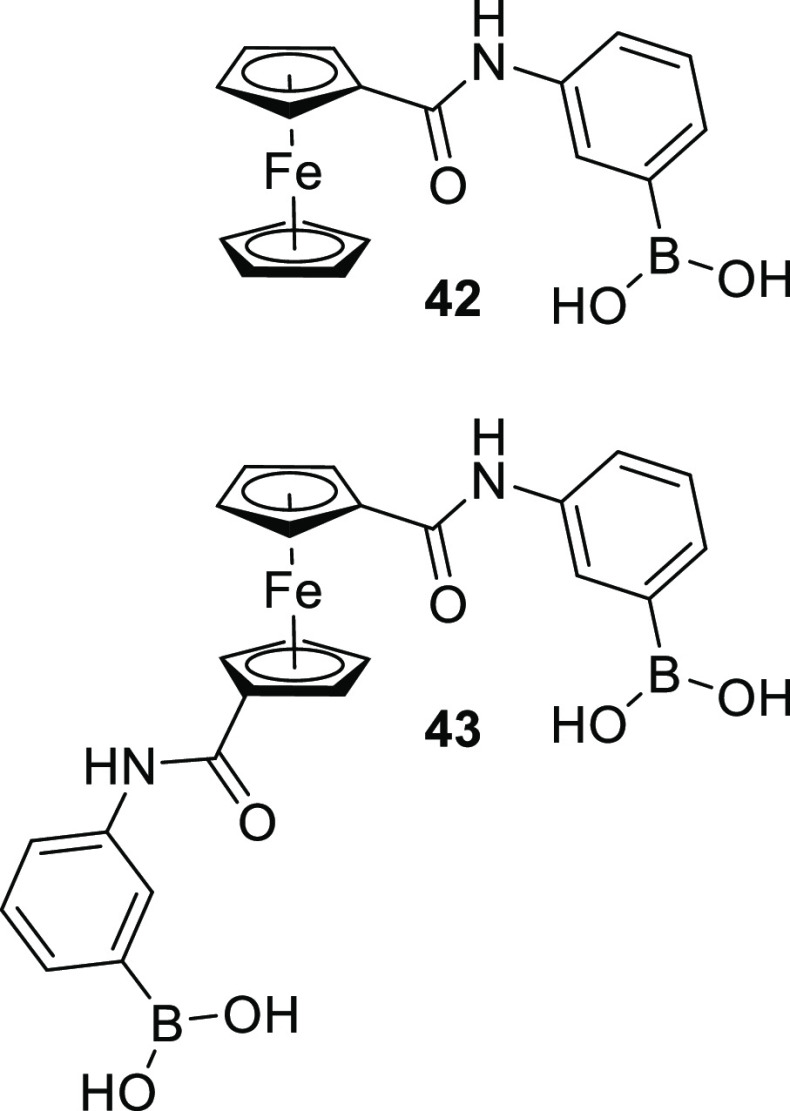
Ferrocene-based electrochemical saccharide sensors **39** and **40** developed by Saleem et al.^130^

While visual responses have advantages such as their ease of use by untrained personnel, sensors with electrochemical outputs offer a much more convenient pathway toward integration into devices. One such example where boronic acid-derived receptors are effectively incorporated into an electrochemical sensing regime was outlined by Saleem et al., who have studied the ability of ferrocene-based boronic acids to detect saccharides.^130^ The researchers synthesized ferrocene (monoaminophenyl)boronic acid **42** as well as 1,1′-disubstituted ferrocene (diaminophenyl)boronic acid **43**, Figure 14, with the ferrocene part acting as a redox reporter in both cases. The researchers initially used proton NMR spectroscopy to confirm the ability the ferrocene (diaminophenyl)boronic acid to form boronic acid:diol complexes with sorbitol. After this had been established, they proceeded to model the electrochemical behaviors of the sorbitol:sensor complexes using cyclic voltammetry. The result was a peak shift in both instances between the bound and unbound forms, indicating the ability of these sensors to detect saccharides. Indeed, the authors demonstrate the ability of the ferrocene (monoaminophenyl)boronic acid sensor to detect the monosaccharides glucose (**1**), fructose (**2**), mannose, and galactose (**3**).

**Scheme 11:**
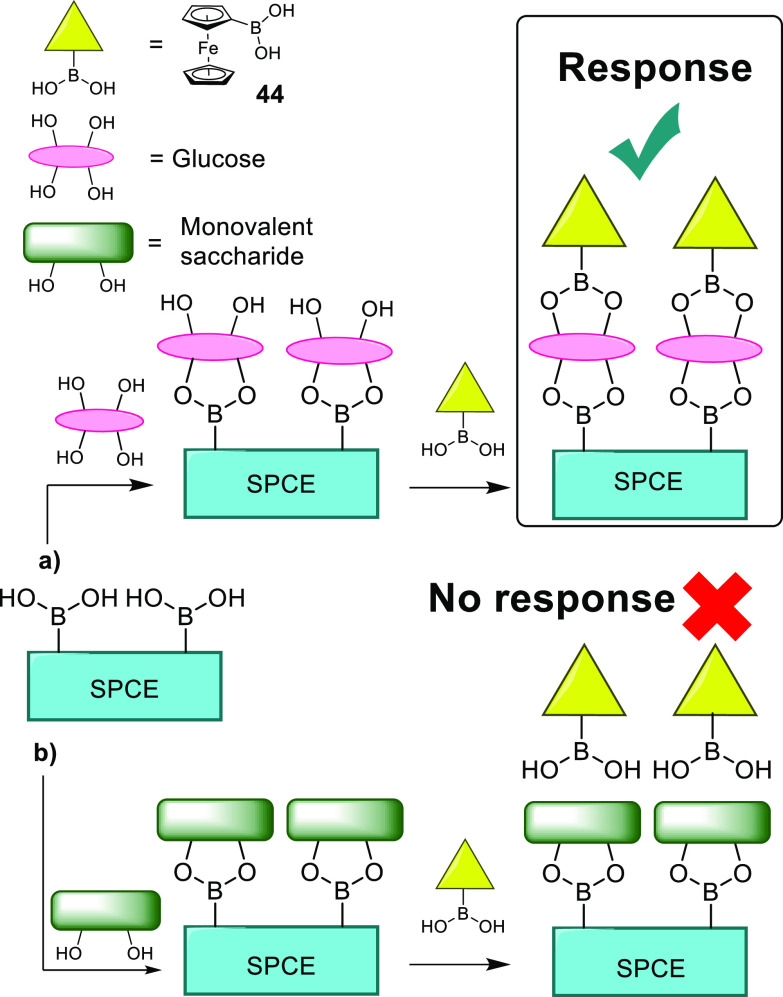
Graphical Representation of How the Sandwich-Type Electrochemical Sensor Developed by Li et al. Offers Selectivity for (a) Divalent Boronic Acid Binder Glucose Forming a “Bridge” and (b) No Response for Other Saccharides Which Only Contain a Single Binding Site

As has been previously discussed, accurate and selective glucose monitoring is essential data used in controlling the impact of diabetes. To this end, Li et al. developed a disposable, sandwich-type electrochemical sensor based on boronic acid derivatives.^131^ They achieved this though the functionalization of a screen printed carbon electrode (SPCE) with ferrocene boronic acid, **44**, creating a surface at which the saccharides could bind. When ferrocene boronic acid was then added, the divalent nature of glucose:boronic acid binding enabled the ferrocene to be bound to the SPCE through the bridging sugar, yielding a current response, Scheme 11. The utilization of the divalent binding gives the system inherent specificity for glucose, which the researchers showed to yield a much greater response than fructose (**2**), mannose, or galactose (**3**). These electrochemical sensors were able to detect glucose at concentrations as low as 0.1 mM at physiological pH, though it was found that the sensitivity increased above pH 8.1, thought to be in accordance with the increased ease of formation of the boronate ester at alkaline pH. To give an indication of the clinical relevance of this system, it was used to measure glucose concentration in urine samples. These samples are often used in the diagnosis of diabetes,^132^ and it was found that the sensor gave accurate and reliable results, despite the complexity of urine as a medium.^131^

**Scheme 12:**
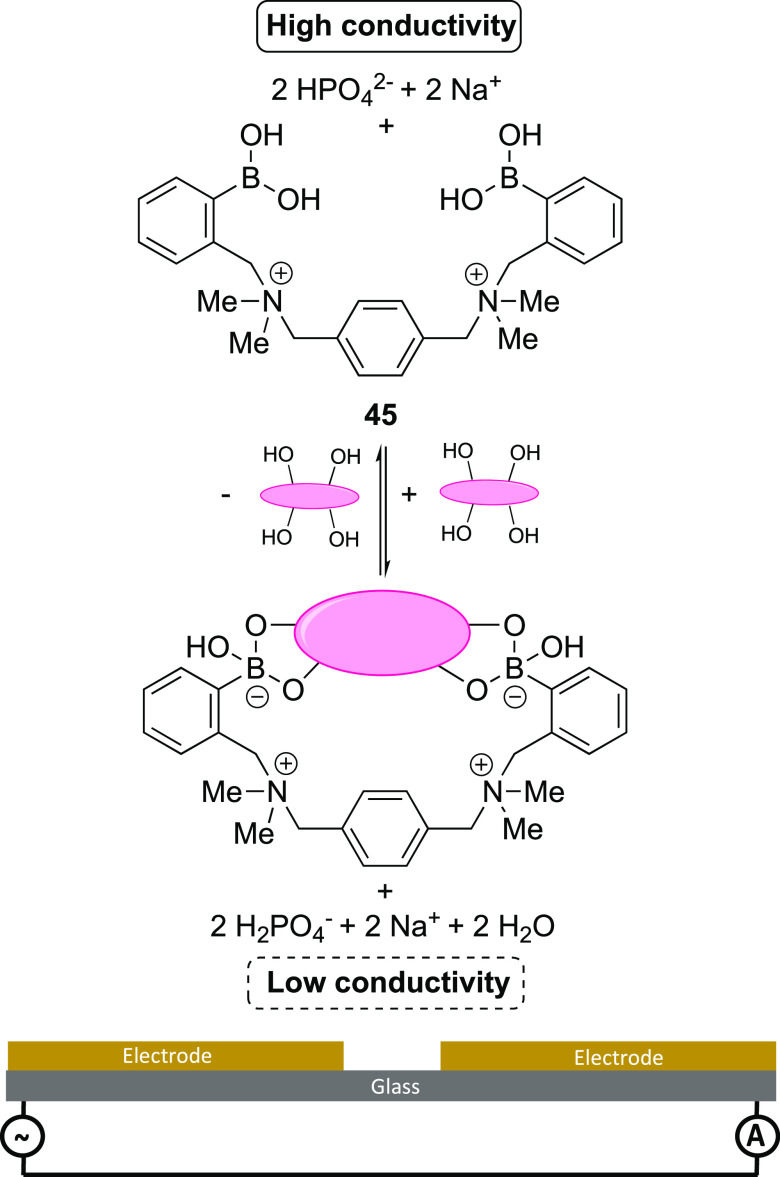
Glucose Detection System Based on Conductivity As Designed by Wang et al.^133^

The p*K*_a_ changes in a boronic acid that occur upon diol binding have been utilized by Wang et al. to produce a highly specific electrochemical sensor for glucose, based on measuring conductivity.^133^ Cationic sensor **45** features two boronic acids held in a rigid structure suitable for glucose complexation, Scheme 12. Upon binding to glucose, the p*K*_a_ of these boronic acids falls from 9.4 to 6.3, causing deprotonation. The authors utilized phosphate buffer, which under these conditions neutralizes the released protons with HPO_4_^2–^, with concurrent formation of H_2_PO_4_^–^. This yielded a measurable decrease in the conductivity of the solution, which could be measured with the use of impedance spectroscopy. The system was found to be specific to glucose (**1**) and fructose (**2**); however, since fructose is physiologically present in such low quantities, the authors determined this interference to be of little relevance to its application in this setting. Indeed, the authors proceeded to test the performance of this sensor in detecting glucose at both physiological (5 mM) and pathophysiological (20 mM) concentrations, in the presence of fructose (**2**), galactose (**3**), lactose (**5**), and maltose (**6**) at three times their maximum plasma concentration. While the presence of fructose caused an ∼3% increase in resistivity when measuring physiological glucose (**1**) concentrations, no other sugar had an effect. This small increase at concentrations triple that of their physiological concentrations indicates the potential for this sensor to be used in CGM.

**Scheme 13:**
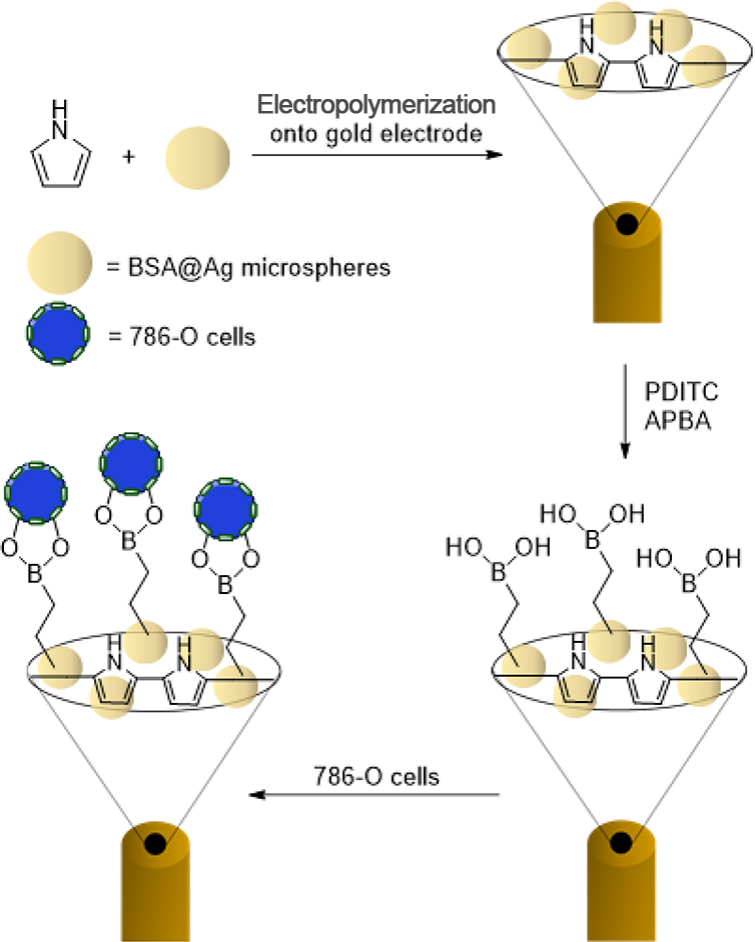
Zhang et al.’s (3-Aminophenyl)boronic Acid Grafted Gold Electrode for Detecting Sialic Acid Using Impedance^134^

The previously outlined ability of boronic acids to form complexes with sialic acid was utilized by Zhang et al. in the development of an electrochemical sensor to aid in the diagnosis of renal cell carcinoma. (RCC).^134^ RCC has a high incidence rate, and current diagnostic techniques are complex and time-consuming, resulting in a diagnosis often occurring when the cancer is already in metastasis.^135^ Sialic acid is overexpressed in RCC cells, making it an attractive target for the development of diagnostic tools. Zhang et al. fabricated Ag@BSA microspheres (gold microspheres coated in bovine serum albumin), which they then formulated into a polypyrrole film. This film was functionalized with (3-aminophenyl)boronic acid, to give the material its sialic acid capturing ability, Scheme 13. This biocompatible film offers a non-invasive method for the detection of sialic acid, and thus RCC (786-O) cells, with a limit of detection of 6 cells mL^–1^, with no response to leukocyte or epithelial cells. Furthermore, the film was evaluated in its ability to detect 786-O cells in the urine of renal cancer patients, as compared to a healthy urine sample. As a further control, 786-O cells were suspended in a sample of healthy urine and this was also tested; it was found that the 786-O doped sample and the samples taken from cancer patients all yielded a response, while the healthy urine did not. This shows the promise of such materials toward novel cancer diagnostics.

**Scheme 14:**
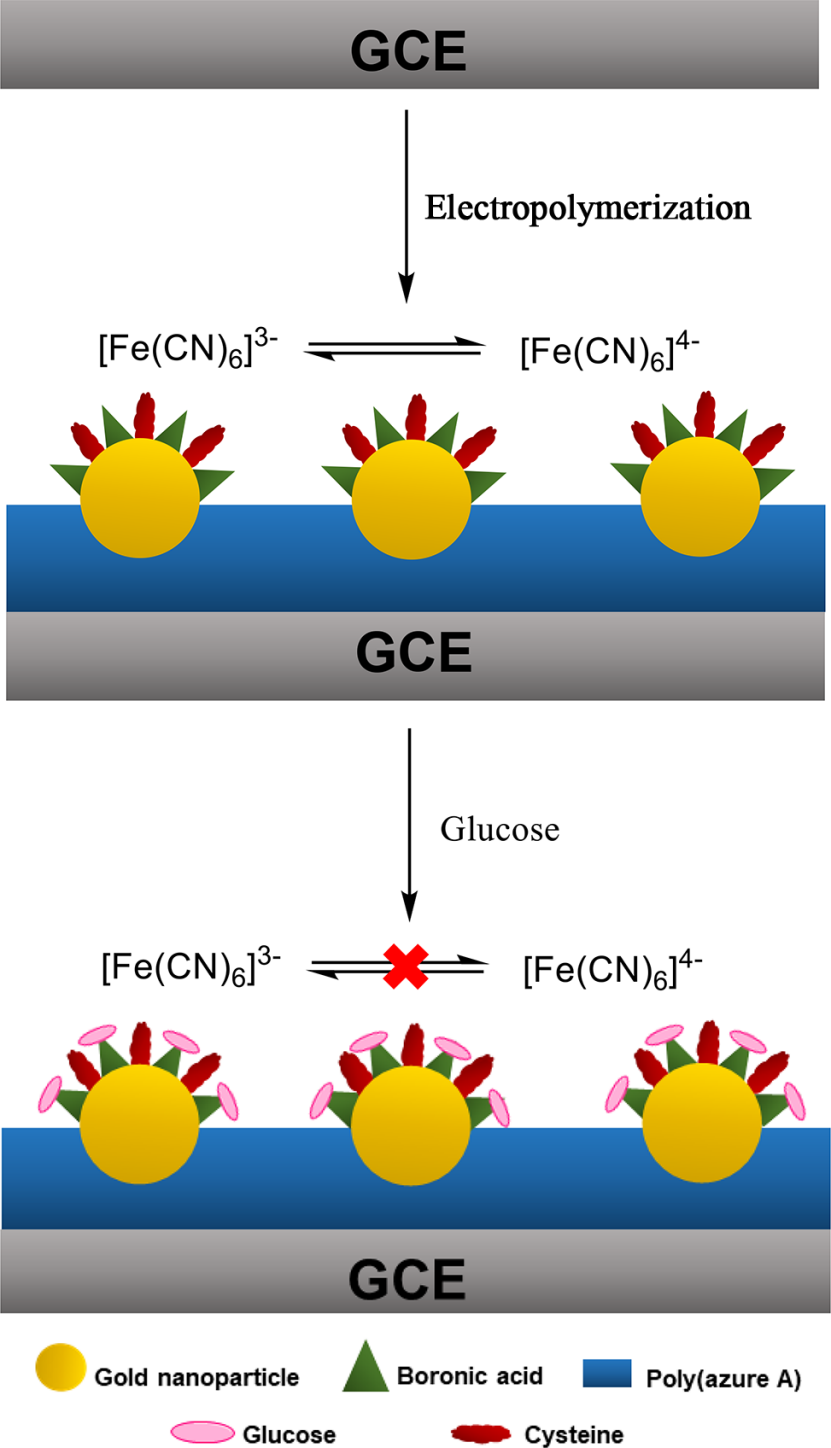
Electropolymerization and Subsequent Glucose Detection by Immobilized Gold Nanoparticles

Toward the development of electrochemical glucose sensors, Wang et al. report the simple one-pot preparation of a composite material that consists of poly(azure A), gold nanoparticles, and 4-mercaptophenylboronic acid, Scheme 14.^136^ The assembly of boronic acid moieties into saccharide sensors can be complex with multiple steps, which has implications when considering large scale production and commercialization. The simplicity inherent in the fabrication of Wang et al.’s system could offer a method of avoiding such potential complications. The polymer was formed using electropolymerization, with simultaneous reduction of HAuCl_4_ to form gold nanoparticles; it was observed that the inclusion of (4-mercaptophenyl)boronic acid appeared to retard the polymerization rate, thought to be due to its weak electroconductivity. Nevertheless, the presence of these functionalized nanoparticles was proven using SEM and FT-IR. Using a ferri-ferroocyanide probe, peak current change was monitored on exposure to glucose. It was shown to be proportional to the logarithm of the glucose concentration at low concentrations (10 nM to 10 μM) with a detection limit as low as 4 nM. The system was also shown to be capable of accurately determining the glucose concentration of diluted human serum samples. Perhaps most importantly, the authors determined that some common physiological contaminants (dopamine, uric acid, and ascorbic acid) had little effect on the sensor’s ability to detect glucose.^136^

**Figure 15:**
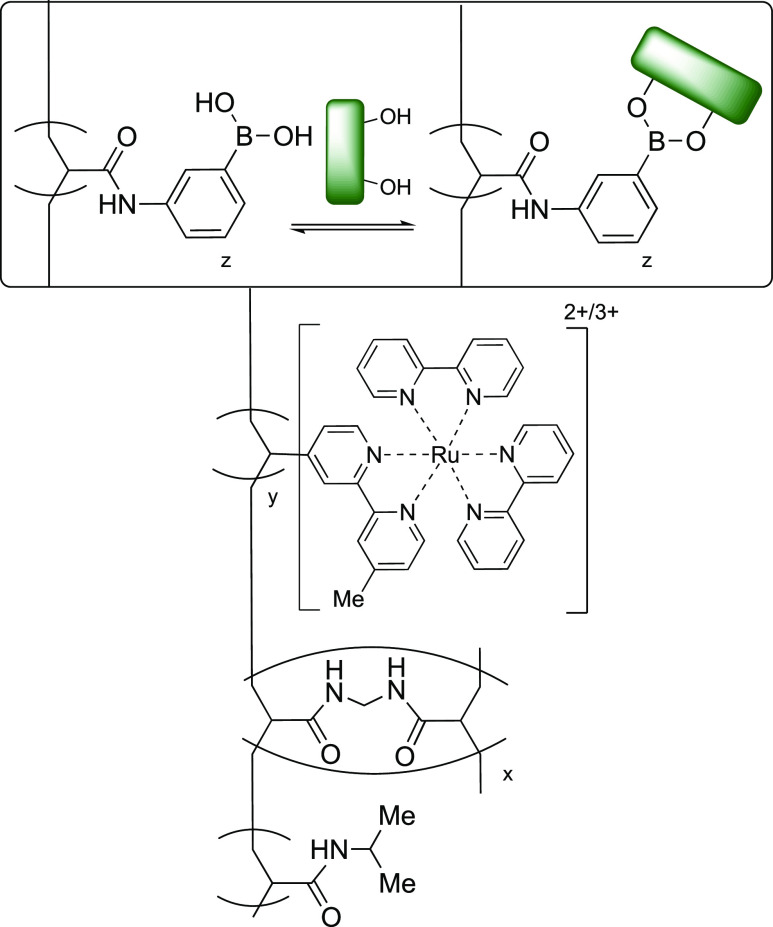
pNIPAM-based polymer containing [Ru(bpy)_3_]^2+^ and PBA exhibiting saccharide responsive chemoluminesence.

Materials that can respond to multiple stimuli have a great variety of uses, with applications in sensing and bioelectronic logic gates. Li et al. have developed a poly(*N*-isopropylacrylamide) (pNIPAM) hydrogel thin film with electrochemical luminescent properties.^137^ pNIPAM has well-documented temperature responsivity; above a certain temperature, defined as the volume phase transition temperature (VPTT), pNIPAM hydrogels decrease in volume. This has led to their use in a diverse range of applications.^138^ The authors covalently bound PBA acid moieties and [Ru(bpy)_3_]^2+^ centers within the pNIPAM matrix, acting as a saccharide-sensing unit and a redox luminophore, respectively, Figure 15. Upon the submersion of the film into solutions of fructose (**2**, 20 mM in PBS, pH 7.4), the PBA forms boronate esters with the fructose. This increase in both hydrophilic character and charge density causes the hydrogel to swell, increasing in thickness from 7 ± 1 to 14 ± 1 μM. The opposite effect was achieved by increasing the temperature from 20 to 40 °C, above its VPTT, provoking film collapse, decreasing the film thickness by a factor of 2. Through cyclic voltammetry, the authors determined the reversible oxidation of the [Ru(bpy)_3_]^2+^ centers to be controlled by charge diffusion within the film; electrons “hop” between the centers; thus, the distance between the redox centers affects the electrochemical luminescence. While the authors did not investigate the sensitivity of this material toward differing concentrations of saccharide, it does provide the outline of a platform for which glucose sensitive materials can be created.

The intrinsic magnetic properties of the 100% abundant spin-1/2 ^19^F nucleus, including high gyromagnetic ratio and no quadrupole moment, make ^19^F NMR spectroscopy a powerful tool for detecting changes in the chemical environment of fluorine-containing molecules, and is increasingly used to interrogate biomolecular processes.^139,140^ The lack of prevalence of fluorine in biological molecules (when compared to hydrogen, carbon, or nitrogen) gives easy to resolve spectra that feature distinct peaks and a high signal-to-noise ratio. In recent years this powerful technique has been used to discriminate and quantify saccharides through their interaction with fluorinated phenylboronic acid derivatives. When considering the use of NMR to study the binding of boronic acids to diols, it may seem obvious to first consider ^11^B NMR. While such studies have been undertaken,^141^ the ^11^B nucleus is quadrupolar, leading to broad peaks which offer poor resolution,^142^ and requires the use of quartz NMR tubes to avoid excessive background signals from borosilicate glass;^143^ the use of ^19^F NMR circumvents these challenges.

**Figure 16:**
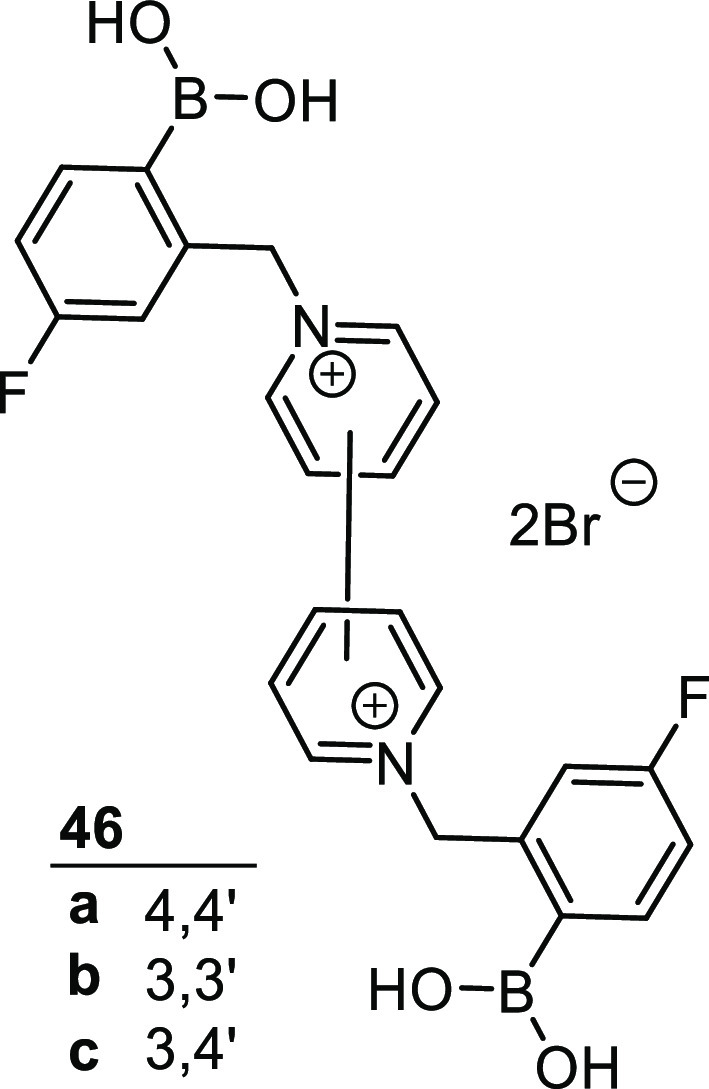
Bispyridinium boronic acids **46a**–**c** used to detect diol binding using ^19^F NMR analysis.

In 2015 Schiller and co-workers reported the successful discrimination of a number of diol-containing saccharides (and other bioanalytes) using ^19^F NMR spectroscopy.^144^ Three water-soluble, fluorinated bisboronic acid bipyridinium salts displaying different substitution patterns were prepared from 4,4′-bipy, 3,3′-bipy, and 3,4′-bipy, **46a**–**c**, Figure 16. Fluorine NMR spectroscopy was then used to monitor the binding of each receptor to each analyte, revealing idiosyncratic spectral shifts as the sp^2^ boronic acids are transformed to the corresponding sp^3^ boronate esters. The equilibrium of the formation of the boron–diol complexes is slow on the NMR time scale, which ensures that the peaks are well-separated. Using a single receptor, discrimination of the analytes was not always conclusive. However, when examined as an array, outputs of the three different channels combined to provide a much more definitive bar-code-type fingerprint.

**Figure 17:**
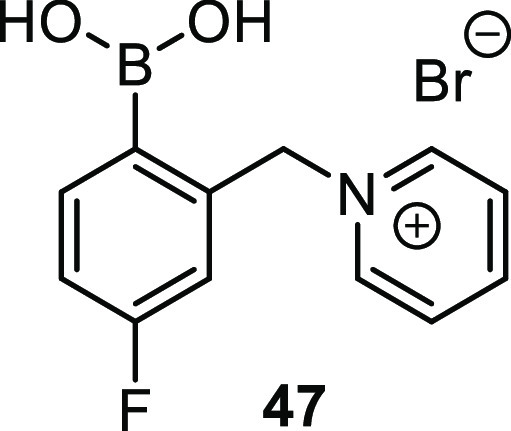
Pyridinium boronic acid **47** used to detect diol binding using ^19^F NMR spectroscopy.

Schiller and co-workers demonstrated the full power of this approach using a single fluorinated monoboronic acid pyridinium salt **47**, Figure 17, to discriminate between a number of biological analytes.^145^

A range of analytes were screened at concentrations of 100 mM d-analyte and 10 mM sensor compound (in HEPES buffer pH 7.4), in order to achieve full receptor saturation. The resulting ^19^F NMR fingerprints were robust to pH changes about the physiological range; the chemical shifts did not vary significantly from pH 6.6 through to 8.2. Glucose (**1**) and fructose (**2**) could be discriminated not only in binary mixtures but also in solutions with up to a 1:9 glucose:fructose molar ratio. To demonstrate the practical utility of this approach, Axlhelm et al. probed the synthetic urine samples and showed this approach to be capable of detecting glucose at concentrations as low as 1 mM.^145^

While this review focuses on primarily small molecular sensors containing saccharide-binding boronic acid (and related) binding motifs, the complementary area of polymeric sensors is also expanding. Particularly noteworthy with respect to the saccharide-selectivity aspects of this review is the area of molecular imprinted polymers (MIPs). A MIP is typically created when a polymer is formed from monomer constituents about an analyte (or analyte surrogate), thus creating an analyte-shaped cavity. While this methodology generally requires stoichiometric access to analyte, the use of boronic acid (and related) monomers^146,147^ in this process can deliver materials with incredibly high spatial and chemical specificity for a chosen saccharide-containing analyte, with features akin to antigen–antibody relationships.^148,149^ Good progress has been made in the detection of glycoprotein biomarkers^150−153^ as well as the detection of smaller saccharides.^154−156^ Combining the benefits of MIPs with molecular chemosensors may offer even more promise for wider ranging applications in the future.

## Conclusion and Outlook

The development of chemosensors for saccharide detection is a research area that has boomed in recent years. In this time, huge advances have been made in the fundamental understanding of the underpinning mechanisms of these sensors, guiding the design of evermore complex boronic acid-containing receptors for an increasing range of analytes. This has also been coupled with novel reporting strategies, with hydrogel-based sensors becoming increasingly prevalent.^157,158^ While there have been great leaps in the field of continuous glucose monitoring,^159,160^ that are helping to overcome the limitations of conventional testing methods,^161^ there remains a need to develop more sensitive, selective, and robust diagnostic tools for saccharides. Selective saccharide recognition, reporting, and sensing molecularly require a well-finessed balance of spatial boronic acid positioning and tuning of subtle electronic parameters. While great strides have been made in this arena, there remains a lack of a unifying platform for sensor discovery and development. Boronic acid derivatives offer the promise of ultimately being one strategy that may address this gap. The works summarized in this review point to a growing foundation that is being built upon by a global community of researchers working toward demystifying the elementary principles of these effects, enabling evermore precise rational design. The requirement for robustness includes stability to ambient conditions such as those experienced during shipping and storage and to biological degradation such as by the unintended action of reactive oxygen species. Research in these areas also contributes toward the development of fundamental understanding that is enabling the development of glucose responsive insulin release technologies,^162−165^ which hopefully will provide quality of life improvements for those with diabetes ahead of a hoped-for cure. As outlined within this review, saccharides have great potential utility as early indicators of cancer, and more recent research is starting to indicate their significance in other disease states.^166^ As these carbohydrate diagnostic targets are identified, so will researchers endeavoring to develop selective boronolectins to study them.

## Vocabulary

Chemosensor: chemosensors are chemical sensors that reversibly bind to an analyte, producing a measurable response, i.e., fluorescence, a color change, or an electrochemical signal
Chemodosimeter: a chemodosimeter is a chemical sensor that irreversibly binds to an analyte, producing a measurable response, i.e., fluorescence, a color change, or an electrochemical signal
Boronolectin: a term to describe sugar-binding receptors that contain boron, particularly boronic acids
Diabetes mellitus: a disease characterized by an inability to regulate blood-glucose levels, resulting in a myriad of harmful effects
Di-, Tri, Oligo-, and Polysaccharides: these are more complex sugars built of two, three, and multiple monosaccharide units, with oligosaccharides traditionally being considered those below ten saccharide units. Polysaccharides are sugar polymers and may consist of a much greater number of individual saccharides, i.e., cellulose, which is formed of repeating units of glucose
Indicator displacement assay (IDA): IDA is a sensing system in which the analyte competes for the binding site of a receptor with an indicator, and in so doing displacement of the indicator by analyte produces a measurable response
